# A Prototype of Graphene E‐Nose for Exhaled Breath Detection and Label‐Free Diagnosis of *Helicobacter Pylori* Infection

**DOI:** 10.1002/advs.202401695

**Published:** 2024-07-04

**Authors:** Xuemei Liu, Qiaofen Chen, Shiyuan Xu, Jiaying Wu, Jingwen Zhao, Zhengfu He, Aiwu Pan, Jianmin Wu

**Affiliations:** ^1^ Lab of Nanomedicine and Omic‐based Diagnostics Institute of Analytical Chemistry Department of Chemistry Zhejiang University Hangzhou 310058 China; ^2^ Will‐think Sensing Technology Co., LTD Hangzhou 310030 China; ^3^ Department of Thoracic Surgery Sir Run Run Shaw Hospital School of Medicine Zhejiang University Hangzhou 310016 China; ^4^ Department of Internal Medicine The Second Affiliated Hospital of Zhejiang University Hangzhou 310003 China

**Keywords:** e‐nose prototype, exhaled breath diagnosis, gas sensors, graphene oxide, *Helicobacter pylori*

## Abstract

*Helicobacter pylori* (HP), a common microanaerobic bacteria that lives in the human mouth and stomach, is reported to infect ≈50% of the global population. The current diagnostic methods for HP are either invasive, time‐consuming, or harmful. Therefore, a noninvasive and label‐free HP diagnostic method needs to be developed urgently. Herein, reduced graphene oxide (rGO) is composited with different metal‐based materials to construct a graphene‐based electronic nose (e‐nose), which exhibits excellent sensitivity and cross‐reactive response to several gases in exhaled breath (EB). Principal component analysis (PCA) shows that four typical types of gases in EB can be well discriminated. Additionally, the potential of the e‐nose in label‐free detection of HP infection is demonstrated through the measurement and analysis of EB samples. Furthermore, a prototype of an e‐nose device is designed and constructed for automatic EB detection and HP diagnosis. The accuracy of the prototype machine integrated with the graphene‐based e‐nose can reach 92% and 91% in the training and validation sets, respectively. These results demonstrate that the highly sensitive graphene‐based e‐nose has great potential for the label‐free diagnosis of HP and may become a novel tool for non‐invasive disease screening and diagnosis.

## Graphical abstract

The detection and analysis of label‐free exhaled breath (EB) of individuals infected with the *Helicobacter pylori* (HP) are performed through a prototype machine integrated with the graphene‐based electronic nose (e‐nose). The analysis results demonstrate that the prototype has a high diagnostic accuracy for HP infection, thereby substantiating its great potential as a new non‐invasive disease screening and diagnostic tool.

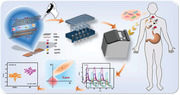

## 1 Introduction

*Helicobacter pylori* (HP) is a spiral‐shaped microaerophilic bacterium first identified in 1983 in gastric mucosal biopsies from patients with gastritis.^[^ ^1^ ^]^ The bacterium colonizes the gastric mucosa, initiating a cascade of inflammatory reactions that can lead to dyspepsia, chronic atrophic gastritis, duodenal ulcers, and possibly even gastric cancer.^[^ ^2^ ^]^ Globally, almost 50% of the population have been infected with HP, with higher rates among those living in developing countries.^[^ ^3^ ^]^ HP infection has been identified by the World Health Organization (WHO) as a primary cause of peptic ulcer disease, gastric muco‐associated lymphoid tissue (MALT) lymphoma, and gastric cancer.^[^ ^4^ ^]^ Numerous studies have shown a direct link between HP infection and stomach cancer rates.^[^ ^5^ ^]^ Infected individuals are reported to have a 3–20 times higher risk of developing gastric cancer than uninfected individuals.^[^ ^4^, ^6^ ^]^ In addition, other gastric and extra‐gastric diseases have also been reported to be associated with HP infection.^[^ ^2^, ^7^ ^]^ Furthermore, on December 21, 2021, the U.S. Department of Health and Human Services (HHS) released its 15th Carcinogenic Report, listing HP as a definite carcinogen. Therefore, the global prevalence of HP and its involvement in the development of numerous severe gastric and extra‐gastric diseases make it imperative to develop rapid, sensitive, and highly specific diagnostic techniques to prevent further transmission of HP.

Clinically, methods for detecting HP can be invasive or noninvasive. Invasive methods include endoscopy,^[^ ^6^ ^]^ histological examinations,^[^ ^8^ ^]^ rapid urease tests,^[^ ^9^ ^]^ and microbial cultures.^[^ ^10^ ^]^ Noninvasive methods include the urea breath test (UBT), the blood antibody test (BAT)^[^ ^4b^ ^]^ and stool antigens tests (SAT).^[^ ^11^ ^]^ Among the various detection methods used in clinical practices, the UBT method is the most commonly used due to its non‐invasive nature and high sensitivity.^[^ ^12^ ^]^ However, this method often needs large equipment, such as mass spectrometry (MS),^[^ ^13^ ^]^ gas chromatography‐mass spectrometry (GC‐MS),^[^ ^14^ ^]^ optical analyzers,^[^ ^15^ ^]^ etc., resulting in complex operation and increased cost. In addition, UBT requires the ingestion of ^13^C or ^14^C labeled urea, which is potentially harmful to human health and the test procedure is time‐consuming.^[^ ^16^ ^]^ Consequently, an ideal non‐invasive method for HP diagnosis is using simple devices to achieve label‐free exhaled breath (EB) analysis.

Convenient and safe label‐free EB analysis is a rapidly growing field with great potential for clinical diagnosis and treatment management. The composition and concentration of human EB reflect the fingerprint of the underlying metabolic and biophysical processes of disease.^[^ ^17^ ^]^ Therefore, EB analysis serves as a non‐invasive diagnostic approach to comprehensively assess a patient's physiological condition.^[^ ^18^ ^]^ Previous studies have reported significant differences in the contents of inorganic gases such as NO^[^ ^19^ ^]^ and NH_3_^[^ ^20^ ^]^ in the EB of HP‐infected people compared to healthy individuals. Specifically, the median concentration of NO in EB is ≈14 ppb in healthy individuals and ≈27 ppb in those infected with HP.^[^ ^21^ ^]^ For NH_3_, in healthy individuals, the concentration in EB is ≈383 ppb. However, in individuals infected with HP, the average concentration is ≈1245 ppb.^[^ ^16^ ^]^ Besides, the infected bacterium also releases volatile organic compounds (VOCs) during metabolic activity. The concentrations of several types of VOCs, such as isoprene,^[^ ^22^ ^]^ styrene, 6‐methyl‐5‐hepten‐2‐one, 2‐propenenitrile, nonanal, and 2‐ethyl‐1‐hexanol,^[^ ^23^ ^]^ as well as 8‐isoprostane and interleukin‐6,^[^ ^24^ ^]^ show differences between HP‐infected and healthy individuals. For instance, healthy individuals typically have isoprene levels in EB ranging from ≈55–121 ppb,^[^ ^25^ ^]^ whereas those infected with HP may have concentrations as high as ≈218 ppb.^[^ ^22^ ^]^ Therefore, label‐free EB detection based on metabolic fingerprint analysis for the noninvasive diagnosis of HP infection is feasible.

Currently, the main technologies used for EB analysis with simple devices include micro GC,^[^ ^26^ ^]^ electronic gas sensors,^[^ ^27^ ^]^ and colorimetric sensors,^[^ ^28^ ^]^ etc. Among them, the chemiresistive sensor is the most promising due to its robust construction and simplicity.^[^ ^29^ ^]^ Nevertheless, a considerable proportion of chemiresistive gas sensors utilize semiconductor metal oxides (SMO) as their sensing components, necessitating elevated operating temperatures,^[^ ^30^ ^]^ thereby resulting in high power consumption and posing limitations on their potential utilization in Internet of Things (IoT) devices. In addition, the sensitivity of SMO is not sufficient to detect ppb‐level trace gases in EB samples. Graphene, a 2D material composed of SP^2^ hybridized carbon atoms, offers distinct advantages in the field of gas‐sensing due to its high specific surface area and exceptional carrier mobility at room temperature.^[^ ^31^ ^]^ Of the numerous graphene‐based sensing material formats, graphene oxide (GO) has attracted considerable attention due to its ease of chemical doping capabilities and surface modification potential, which is attributed to its rich oxygen‐containing functional groups.^[^ ^32^ ^]^ Previously, we demonstrated that the introduction of poly (diallyl dimethylammonium chloride) (PDDA) can significantly improve the gas detection sensitivity of reduced graphene oxide (rGO).^[^ ^33^ ^]^ In addition to the requirement of high sensitivity, excellent gas selectivity is also essential for identifying feature gases in EB samples. Due to the inherent constraints of the sensing mechanism, achieving absolute selectivity in chemiresistive sensors for detecting specific gas analytes remains a challenging task. To address the problem, a sensing array with a cross‐reactive response to different types of gases is usually employed.^[^ ^34^ ^]^ Taking into account the sensitivity and cross‐reactive selectivity, designing a combination of active receptors in rGO‐based chemiresistive sensing arrays for EB discrimination is highly demanded. Meanwhile, the interference of humidity is another challenge in EB analysis since the chemiresistance response caused by humidity can mask the true signal of the feature gas components in EB. Therefore, it is also crucial to design dehumidification methods and equipment for EB analysis.

Herein, we introduce a variety of metal‐based materials to rGO as different types of active receptors since they have different sensing mechanisms and selectivity to bind with feature gases involved in the metabolic processes of the human body and infected bacteria. With the assistance of PDDA, a polycationic molecule, different types of metal‐based materials were composited with rGO to construct an e‐nose array (rGO‐PDDA‐M).^[^ ^33^ ^]^ The PDDA can not only improve the gas sensitivity of the sensor, but also prevent rGO aggregation during liquid phase reduction. The rGO‐PDDA‐M sensor array exhibits excellent gas sensing sensitivity and selectivity in detecting typical trace gases in EB. Meanwhile, the e‐nose displays high discrimination ability for four types of feature gases including nitric oxide (NO), ammonia (NH_3_), isoprene (Iso) and acetone (Ace) in PCA. Due to the high correlation of these feature gases in the metabolic pathway of infected HP, the graphene e‐nose shows high accuracy in label‐free HP diagnosis after dehumidification with a cooling device. Moreover, a compact microsensor chip and an e‐nose prototype device were designed and constructed for EB analysis. The lasso regression pattern recognition algorithm was used to distinguish EB samples of HP‐positive patients from healthy individuals with 92% accuracy, 93.1% sensitivity and 97.4% specificity. This work demonstrates that the prototype of the EB analyzer integrated with the rGO‐PDDA‐M microsensor has great potential for HP label‐free analysis and non‐invasive clinical diagnosis.

## 2 Results and Discussion

### 2.1 Screening of Sensing Materials for the rGO‐PDDA‐M Sensing Layers

Although rGO is a popular carbon‐based gas‐sensitive material that has garnered extensive research focus in recent years, there is still significant room for improving its gas detection sensitivity and selectivity.^[^ ^31b^ ^]^ Our previous works have shown that metal ions and TMDs doped rGO‐based hybrids can effectively enhance gas‐sensitive properties by introducing abundant metal highly active sites for gas adsorption.^[^ ^33^, ^34^ ^]^ In addition, the decoration of noble metals can contribute to electron transfer effects in nanocomposites and introduce highly active catalytic sites, which exactly pave a new extraordinary road for the enhancement of gas sensing performances.^[^ ^35^ ^]^ Furthermore, our previous work^[^ ^33^ ^]^ has demonstrated that the polycationic electrolyte PDDA can be employed to assemble MoS_2_ and rGO and significantly improve gas sensing sensitivity. As GO is not conductive, a reduction process is required to test its resistance response signal. In the previous work, the gas phase reduction method was employed. However, the liquid phase reduction technique was adopted for the sake of operational simplicity in the present work. In addition, PDDA could prevent the aggregation of rGO when GO was reduced in an aqueous solution. Otherwise, obvious aggregation of rGO in the liquid phase could be observed (Figure S1a, Supporting Information). In contrast, when PDDA was added, the rGO‐PDDA presented a uniform suspension (Figure S1b, Supporting Information). The aggregation of rGO could result in the deterioration of gas sensing performance. As shown in Figure S2a,b (Supporting Information), the rGO‐PDDA exhibited higher sensitivity and lower electrical noise compared to the rGO. In addition, the stability test over a 15‐day period indicated that rGO‐PDDA exhibited better stability compared to pure rGO (Figure S2c, Supporting Information). Consequently, PDDA was still introduced into the composites in this study. Specifically, 14 types of metal‐based composites, including metal nanoparticles, 2D‐MoS_2_, metal ions and their combinations, were screened based on their sensitivity and cross‐reactive responses to NO and isoprene, in which NO is the most sensitive gas while isoprene is a typical organic gas in HP‐infected EB samples. As indicated in Figure S3 (Supporting Information), rGO‐PDDA‐M exhibited much higher gas sensitivity compared to rGO. This can be attributed to the introduction of metal‐based receptors, which enhanced the binding and adsorption ability between target gases. Eventually, eight sensing layers including rGO‐PDDA‐Co/Fe, rGO‐PDDA‐Co/Cu, rGO‐PDDA‐Au/Ag, rGO‐PDDA‐Ag, rGO‐PDDA‐MoS_2_, rGO‐PDDA‐Ce, rGO‐PDDA‐Fe, and rGO‐PDDA‐Co were selected to construct the sensor array duo to their higher sensitivity, low baseline noise (Table S1, Supporting Information) and cross‐reactive response toward isoprene, and NO (Figure S3, Supporting Information).

### 2.2 Characterization of Different Sensing Materials

**Figure 1:**
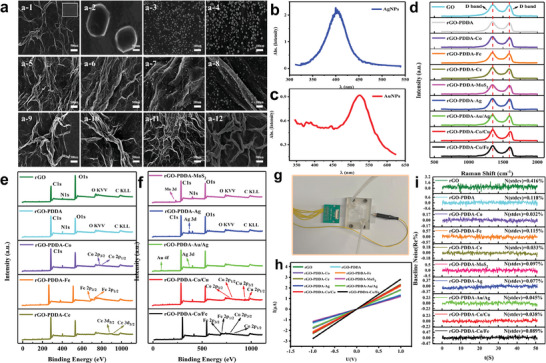
SEM pictures of different materials. a‐1) rGO (insert in (a‐1)) and rGO‐PDDA; a‐2) MoS_2_; a‐3) AuNPs; a‐4) AgNPs; a‐5) rGO‐PDDA‐Co/Fe; a‐6) rGO‐PDDA‐Co/Cu; a‐7) rGO‐PDDA‐Au/Ag; a‐8) rGO‐PDDA‐Ag; a‐9) rGO‐PDDA‐MoS_2_; a‐10) rGO‐PDDA‐Ce; a‐11) rGO‐PDDA‐Fe; a‐12) rGO‐PDDA‐Co. The UV–vis extinction spectrum of b) AuNPs; c) AgNPs; d) The Raman spectra of different materials; e,f) The XPS spectra of different materials. g) The 8‐channel sensing array chip. h) The pictures of *I*–*V* curves of different materials on electrodes; i) The baseline noises of different materials on electrodes.

**Figure 2:**
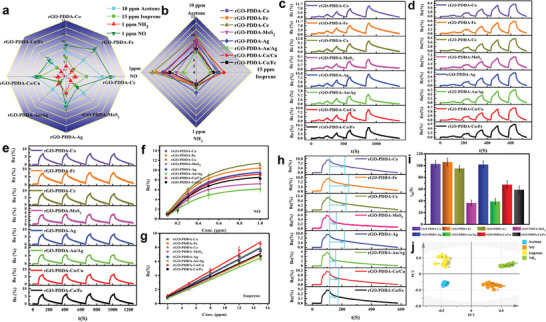
The radar of response values of rGO‐PDDA‐M sensor; a) different composites; b) different gases; Response curves of rGO‐PDDA‐M to c) 0.1‐1 ppm NO; d) 2–15 ppm isoprene; e) Response repeatability of rGO‐PDDA‐M to 10 ppm isoprene. Langmuir fitting of rGO‐PDDA‐M toward f) NO; g) Linear fitting of isoprene; h) Response dynamic curves of rGO‐PDDA‐M to 1 ppm NO (The blue highlight was t_50_ of each sensing layer); i) The t_50_ values of rGO‐PDDA‐M to 1 ppm NO; j) PCA analysis conducted based on the normalized Re% and t_50_ of four typical gases.

**Figure 3:**
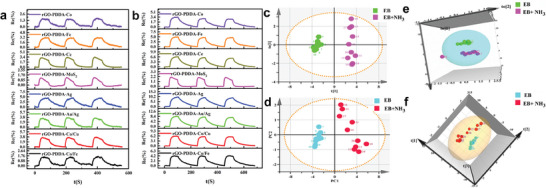
Response dynamic curves of rGO‐PDDA‐M sensor array toward EB sample of a) pure EB; b) spiked EB (1 ppm NH_3_). c) 2D OPLS‐DA; d) PCA e) 3D OPLS‐DA and f) PCA results according to Re% of rGO‐PDDA‐M sensor array toward pure EB and spiked EB (1 ppm NH_3_).

**Figure 4:**
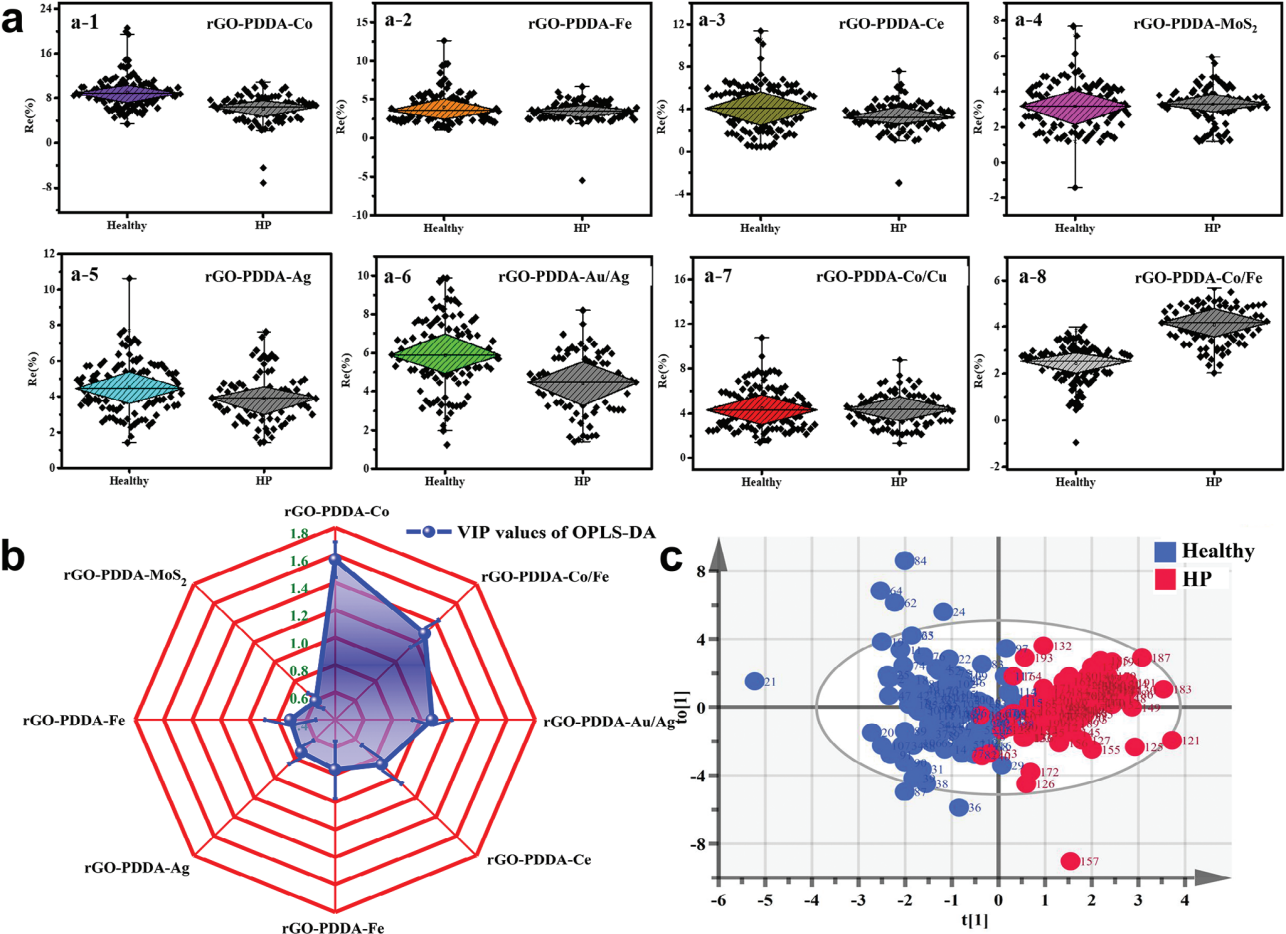
a) Box charts of response values of clinical EB samples; (a‐1‐a‐8) Response values of rGO‐PDDA‐Co; rGO‐PDDA‐Fe; rGO‐PDDA‐Ce; rGO‐PDDA‐MoS_2_; rGO‐PDDA‐Ag; rGO‐PDDA‐Au/Ag; rGO‐PDDA‐Co/Cu; and rGO‐PDDA‐Co/Fe toward EB samples of healthy person (colored) and people infected with HP (black); b) Radar map of VIP values of Re% variable of clinical EB samples analysis at 8 sensing layers of the sensor array; c) The OPLS‐DA result of clinical EB samples analysis at 4 sensing layers of the sensor array. (*n* = 204; α = 0.05; *P* < 0.05; n is sample size; α is the level of significance test; and the P value is the probability that if the null hypothesis is true, an outcome more extreme than the obtained sample observations will occur.).

The morphologies of pure GO, MoS_2_, AuNPs, AgNPs, and rGO‐PDDA‐M were observed by SEM (**Figure** **1**a). The SEM images revealed that the rGO‐PDDA and rGO‐PDDA‐M exhibit a rougher, more undulating, and porous surface texture in comparison to the pristine rGO (insert in Figure 1a‐1). Lamellar sizes of ≈1um could be observed in the picture of MoS_2_ (Figure 1a‐2), and the rGO‐PDDA‐MoS_2_ hybrid exhibited a folded flower‐like structure (Figure 1a‐9). Besides, the SEM images indicated that the particle size of the AuNPs is ≈10–20 nm, while the size of the AgNPs is ≈10–30 nm (Figure 1a‐3,a‐4). A successful combination of Au/Ag NPs and rGO‐PDDA was also observed (Figure 1a‐7,a‐8). Moreover, the UV–vis spectra exhibited that AuNPs and AgNPs had a characteristic peak at ≈525 and ≈405 nm (Figure 1b,c), respectively. In the light of these empirical relationships^[^ ^36^ ^]^ and combined with the SEM results above, it can be reasonably inferred that the particle dimensions of the prepared AuNPs and AgNPs are ≈10–20 and ≈10–30 nm, respectively.

Measurements of the Raman spectra were conducted on the rGO‐PDDA‐M hybrids positioned on a glass substrate. For the GO and rGO‐PDDA, both D‐band at ≈1345 cm^−1^ and G‐band situated at ≈1590 cm^−1^ were found (Figure 1d). The D‐band was attributed to the presence of disordered C atoms and defects, whereas the G‐band signified the vibration of ordered sp^2^ C atoms within a 2D hexagonal lattice. The D/G intensity ratios observed in the Raman spectra of rGO‐PDDA and rGO‐PDDA‐M hybrids are higher than those of GO, suggesting an increase in the disorder of carbon atoms. The XPS analysis of rGO‐PDDA and rGO‐PDDA‐M revealed the presence of C and O elements. Weak Co 2p, Fe 2p, Cu 2p, Ce 3d, Au 4f, and Ag 3d peaks at ≈790, ≈720, ≈940, ≈900, ≈85, and 375 eV can be observed in rGO‐PDDA‐M composites compared with rGO and rGO‐PDDA, respectively (Figure 1e,f). In the high‐resolution C1s images of rGO, rGO‐PDDA, and rGO‐PDDA‐M samples, four distinct peaks were observed, corresponding to ≈284.5 eV (C═C, C─C, C─H), 285.5 eV (C─OH, C─N, or other similar bondings), 286.5 eV (C─O─C), 288 eV (C═O), and 289.2 eV (O═C─O),^[^ ^37^ ^]^ suggesting that the original structure of rGO was still preserved in the rGO‐PDDA‐M composites (Figure S4, Supporting Information). A peak at ≈283.4 eV could be attributed to C─M bonds in the rGO‐PDDA‐M composite.^[^ ^34^ ^]^ Furthermore, the valence state of metal elements in rGO‐PDDA‐M composites was further analyzed by XPS high resolution scanning technology. As observed in Figure S5a (Supporting Information), Co element existed in the form of Co^2+^(2p_3/2_ 784.8 and 2p_1/2_ 802.7 eV) and Co^3+^(2p_3/2_ 780.9 and 2p_1/2_ 796.7 eV).^[^ ^38^ ^]^ Fe existed in the states of Fe^2+^ (2p_3/2_ 711.1 and 2p_1/2_ 724.5 eV) and Fe^3+^(2p_3/2_ 713.9 and 2p_1/2_ 726.6 eV),^[^ ^39^ ^]^ and two satellite peaks located at 718.8 and 733.4 were observed (Figure S5b, Supporting Information). Besides, the characteristic peaks at 934.5 and 953.4 eV were classified as Cu^+^, while the peaks at 940.3 and 953.3 eV were attributed to Cu^2+^ (Figure S5c, Supporting Information).^[^ ^40^ ^]^ It was worth noting that the peaks at 944.5 and 963.1 eV belonged to the satellite peaks of the Cu element. Regarding the Ce element, the entire spectrum was decomposed into two bands: the v and u bands, which are associated with the 3d electrons of Ce occupying the spin‐orbital states of 5/2 and 3/2, respectively. Specifically, the peaks at 917.1, 899.4, 903.9, and 885.6 eV were assigned to Ce^3+^ state, which were marked separately as v_0_, u_0_, v’, and u’ (Figure S5d, Supporting Information).^[^ ^41^ ^]^ Other peaks correspond to Ce^4+^ and were also labeled as v (906.5 eV), u (887.9 eV), v’’ (901.6 eV), u’’ (883.4 eV), v’’’ (898.1 eV), and u’’’ (881.1 eV).^[^ ^42^ ^]^ And the two main characteristic peaks at 232.3 and 235.5 eV were attributed to Mo 3d_5/2_ and Mo 3d_3/2_ (Figure S5e, Supporting Information), respectively. High‐resolution spectra of the Ag 3d peak measured in the rGO‐PDDA‐Ag composite showed two peaks at ≈368.1 eV (Ag 3d_5/2_) and ≈374.2 eV (Ag 3d_3/2_) (Figure S5f, Supporting Information).^[^ ^29^ ^]^ The orbital interaction splitting of the 3d doublet was 6.0 eV, confirming the existence of Ag NPs. Moreover, the high‐resolution spectra of Au 4f and Ag 3d revealed the coexistence of AuNPs and AgNPs in the rGO‐PDDA‐Au/Ag composite (Figure S5g,h, Supporting Information).^[^ ^43^ ^]^ It should be noted that, compared with pure AuNPs and AgNPs, the binding energy of the characteristic peak of noble metals shifted to a higher binding energy (Figure S5f–j, Supporting Information). Specifically, during the three‐cycle tests under vacuum, the average binding energy for Ag 3d_5/2_ increased from ≈366.8 to ≈368.1 eV, and for Ag 3d_3/2_, it increased from ≈372.7 to ≈374.2 eV. For Au 4f, the values increased from ≈82.6 (Au 4f_7/2_) to ≈83.8 eV and from ≈86.4 (Au 4f_5/2_) to ≈87.7 eV, respectively. This shift was caused by the electron transfer between the noble metals and rGO due to the difference in their work functions.^[^ ^35^ ^]^

After 8 types of rGO‐PDDA‐M were loaded on the 8‐channel ITO‐PET interdigital electrodes (IDEs), the sensor chip was placed into the lab‐made gas tank (Figure 1g) and the current‐voltage (*I*–*V*) curves were tested (Figure 1h). All sensing elements exhibited stable linear *I–V* curves, indicating ohmic contact behavior between the composites and electrodes. Concurrently, the electrical noise in the rGO‐PDDA and rGO‐PDDA‐M was lower than that of pure rGO reduced by NaBH_4_ (Figure 1i), suggesting more stable contact with electrodes for the rGO‐PDDA and rGO‐PDDA‐M.

### 2.3 Standard Gas Sensing Performance of the rGO‐PDDA‐M Sensor Array

Four typical organic and inorganic gases in EB including acetone, isoprene, NO and NH_3_ were selected for our study. The Re% of the four typical gases and dynamic response curves toward NO and isoprene of each sensing layer of rGO‐PDDA‐M array were shown in **Figure** **2**a–d, respectively. The radar maps indicate that the rGO‐PDDA‐M sensor array displayed cross‐response to four typical gases, attributing to the introduction of different metal‐based elements. Good repeatability was manifested in multiple cycles of isoprene, acetone, NO and NH_3_ (Figure 2e; Figure S6, Supporting Information). Besides, the correlation between response signals and the concentration of (four typical gases) was further analyzed. For acetone, NO and NH_3_, the Langmuir model could be fitted well, while a significant linear relationship was found between the Re% and the concentration of isoprene (Figure 2f,g; Figure S7a,b, Supporting Information). The fitting parameters were listed in Tables S2 and S3 (Supporting Information). Meanwhile, the limit of detection (LOD) of rGO‐PDDA‐Co/Fe site for NO and NH_3_ can reach as low as 6 and 16 ppb (3δ/S), respectively, showing a significant advantage over previously reported rGO‐based NO and NH_3_ gas sensors (**Table** **1**). In addition, Table S4 (Supporting Information) shows that the LOD of the rGO‐PDDA‐M sensor array reaches the minimum concentration required to detect HP, demonstrating the potential of the array to diagnose HP infection through EB analysis. Furthermore, the responses of the sensor array toward CO, NO_2_, and H_2_S usually present in EB were also measured, that all had high sensitivity and the Re% can reach more than 10% at a concentration of 1 ppm (Figure S8, Supporting Information), further confirming the high sensitivity of the graphene‐based array and potential for use in EB analysis.

**Table 1:** Comparison of gas sensing performance with previously reported rGO‐based materials.

Materials	Gas	Temperature	LOD
rGO/PDDA/Co_3_(HITP)_2_^[^ ^44^ ^]^	NO	RT	11 ppb
WS_2_/MWCNT^[^ ^45^ ^]^	NO and NH_3_	18	5 ppb for NO 100 ppb for NH_3_
Ti_3_C_2_Tx/SnS_2_^[^ ^46^ ^]^	NH_3_	18	10 ppb
Cd doped ZnO^[^ ^47^ ^]^	NH_3_	RT	5 ppm
ZnIn_2_S_4_/ Cu_2_O^[^ ^48^ ^]^	NH_3_	RT	15 ppm
Co_3_O_4_@PEI/Ti_3_C_2_T^[^ ^49^ ^]^	NO	RT	30 ppb
Nanostructured WO_3_ film^[^ ^50^ ^]^	NO	250	4.3 ppb
Sputtering WSe_2_^[^ ^51^ ^]^	NO	250	24 ppb
rGO‐PDDA‐Co/Fe in this study	NO and NH_3_	RT	6 ppb for NO 16 ppb for NH_3_

To evaluate the gas discrimination ability of the sensor array, PCA analysis was performed with Re% as the characteristic value, the results displayed a good distinction between the four typical gases, but there was still room for improvement in distinguishing between acetone and isoprene (Figure S7c, Supporting Information). To enhance the gas discrimination ability, another characteristic value, t_50_ (half recovery time) of each site was additionally introduced. Each sensing material has specific adsorption kinetics, thereby resulting in different t_50_ values for each gas at each site (Figure 2h–i; and Table S5, Supporting Information). Taking both Re% and t_50_ as the characteristics input value, the rGO‐PDDA‐M array shows excellent ability to distinguish the four typical gases including acetone, isoprene, NO and NH_3_ (Figure 2j). The results suggested that more input data collected from the sensing curve can reveal the feature response of specific types of gases.

### 2.4 Mechanism Study

In our previous work, the response mechanisms of the rGO and rGO‐PDDA have been discussed.^[^ ^33^ ^]^ In this study, the addition of metal‐based receptors introduces specific sites to enhance gas adsorption and further increase sensitivity. Besides, the porous and rough structure morphology resulting from the addition of metal‐based materials could facilitate gas diffusion onto the surface of sensing materials (Figure 1a).^[^ ^33^, ^34^, ^52^ ^]^ Specifically, the mechanisms of the chemiresistance sensor include electron transfer mechanism and electron tunneling hopping mechanism dominated by interparticle distance and permittivity.^[^ ^34^, ^53^ ^]^ For rGO‐PDDA‐M composites incorporated with Co, Fe, Ce, and Cu ions, the interlayer space of the composites themselves is dominated by the intercalated PDDA, since the dimension of ions is much smaller than that of PDDA. When composites are exposed to sensing gases, the transitional metal ions play the role of active sites for gas adsorption. The adsorbed gas causes a change in interlayer spacing and permittivity between the rGO layers, thereby leading to a change in electron tunneling capability and overall conductivity. Therefore, in rGO composites containing metal ions, the electron tunneling hopping mechanism plays a dominant role. In contrast, when AuNPs and AgNPs are incorporated into rGO‐PDDA‐M, the mismatch in work functions leads to a primary transfer of carriers between the noble metal nanoparticles and rGO. This, in turn, enhances the thickness of the depletion layer, thereby sensitizing its conductivity response.^[^ ^29^ ^]^ As mentioned in Section 2.2, the XPS spectra of the composites show a shift in the binding energy of Au 4f and Ag 3d to a higher binding energy, providing direct evidence of this charge transfer (Figure S5f–j, Supporting Information). Moreover, the incorporation of AuNPs and AgNPs promotes the dissociation of O_2_, resulting in a greater number of chemisorbed O^2−^. Later, these ions spill out onto the surface of the composites, reacting with a good deal of target analytes, ultimately enhancing their overall gas sensing capabilities.^[^ ^35^ ^]^ Therefore, for rGO‐PDDA‐M containing AuNPs and AgNPs, in addition to the electron tunneling hopping mechanism, the electron transfer mechanism has a great influence and may even dominate the whole mechanism. To further prove it, we tested the sensing behaviors of different rGO‐PDDA‐M composites toward different types of gases. As acetone and isoprene lack significant oxidizing or reducing properties, the responses of acetone and isoprene should be mainly attributed to electron tunneling mechanism. The results confirmed that the response of sensing materials incorporated with noble metal nanoparticles is lower than that of metal ions (Figure 2a,b). Furthermore, due to the higher dielectric constant of acetone compared to isoprene, the rGO‐PDDA‐M exhibits higher response values to acetone than to isoprene. In the case of typical inorganic gases such as NH_3_, NO_2_, and NO, both electron transfer and tunneling mechanisms exist. For the NH_3_ gas with electron‐donating ability, the electron transfer mechanism resulted in a decrease of conductivity due to the lowing of the majority charge carrier (hole in p‐type rGO^[^ ^32^, ^54^ ^]^), while the electron tunneling mechanism caused the swelling of the interlayer and consequently the decrease in conductivity. Therefore, the responses of rGO‐PDDA‐M containing noble metal nanoparticles are generally larger than those of metal ions (Figure 2a,b). For NO_2_ with high electron accepting ability, the electron transfer mechanism results in an increase in the conductivity of composites due to the increase in the majority charge carrier, causing a downward resistance response signal. In the electron tunneling hopping mechanism, when the NO_2_ gas passes through the composites, the adsorption of gas causes the expansion of interlayer spacing of rGO, resulting in a reduction of electron hopping ability between the rGO layers and ultimately leading to an upward resistance response signal. Therefore, the mechanisms involved in the responses to NO_2_ result in opposite effects and partially counteract each other. In addition, the modified and doped graphene has more vacancy defects on its surface compared to rGO, causing the strong chemisorption of NO_2_.^[^ ^55^ ^]^ The chemi‐adsorbed NO_2_ molecule has a binding energy of −6.411 eV and a dissociation energy of 305 kJ mol^−1^,^[^ ^56^ ^]^ which is much higher than that on the rGO surface. Accordingly, the rGO decorated with metal receptors displays a significantly higher response than that on the rGO. Meanwhile, the response caused by the electron transfer mechanism on the rGO‐PDDA‐M could more significantly offset the response caused by the electron tunneling hopping mechanism. For the noble metal nanoparticle decorated rGO, its electron transfer mechanism is more obvious. As a result, it can compensate for the increased resistance response caused by the electron tunneling hopping mechanism. Therefore, composites containing noble metal nanoparticles produce a lower response than composites incorporated with metal ions (Figure S8b, Supporting Information).

In contrast to NO_2_, which is a strong oxidizing gas, NO is reductive in this system. Therefore, in the electron transfer mechanism, NO gas produces an upward resistive response. Furthermore, as NO is partially oxidized to NO_2_, which in turn produces a downward resistive response, the two types of responses partially cancel each other out in the electron transfer mechanism. Consequently, the response of NO is mainly based on the electron tunneling hopping mechanism (Figure 2a,b). Moreover, compared to AuNPs, the catalytic performance of AgNPs is much higher, thereby greatly accelerating the kinetics of surface electron transfer.^[^ ^29^, ^57^ ^]^ As a result, rGO‐PDDA‐Ag exhibits higher gas sensitivity to typical gases compared with rGO‐PDDA‐Au (Figure S3, Supporting Information). However, the sensitivity of rGO‐PDDA‐Au/Ag was relatively low (Figure S3, Supporting Information), which may be attributed to the fact that AuNPs can act as catalyst for AgNPs deposits, which cause excessive accumulation of nanoparticles on the surface of rGO and result in metallic‐like materials, thereby reducing the gas sensing performance of the composite.^[^ ^58^ ^]^

### 2.5 Method Optimization for EB Detection

Given the considerable concentration of CO₂ in human exhaled breath, the response of the rGO‐PDDA‐M sensor array to CO₂ was also studied. As shown in Figure S9 (Supporting Information), the response values of each site of the sensor array to 1000 ppm CO_2_ were only ≈0.2%, indicating a much lower response to CO_2_ compared to other target gases. Despite this, the concentration of CO_2_ in EB is ≈4%, which remains a notable signal in multi‐cycle tests (Figure S10, Supporting Information). However, as the concentrations of CO_2_ are nearly identical from person to person, the responses generated by CO_2_ will not impact the normalized response pattern used to differentiate between healthy and HP patients.^[^ ^33^ ^]^ Nevertheless, the humidity of EB significantly affects the response pattern of the sensor because the sensor array is highly sensitive to humidity (Figure S11, Supporting Information). To study the response of the rGO‐PDDA‐M to moisture, the EB sample of a healthy volunteer was collected and tested using the method outlined in Section “EB Sample Collection and Measurement Method”. The results showed that the response signals of each sensing layer were almost the same since they were largely obscured by the humidity (Figure S12, Supporting Information). Therefore, we attempted to use the condensation method described in Section “EB Sample Collection and Measurement Method” to avoid or alleviate the influence of humidity in EB analysis. As exhibited in Figure S13 (Supporting Information), a nine‐grid ice pack is packed with insulated paper into a cylinder shape, and then a Teflon tube is passed through the cylindrical condensing pipe for the EB samples to flow through. The response curves of the sensor array to the EB sample were retested. We can observe that, after such a cooling condensation process, the response of each sensing element in the sensor array was significantly reduced due to the reduction of interference from humidity, and obvious differences between each site were observed (Figure S14, Supporting Information). The results suggested that the condensation process to reduce moisture was essential for the EB sample analysis. To further confirm the reliability of the dehumidification method described above, 10 cases of EB sample collected from healthy individuals were measured. Each sensing layer also exhibited significant differences in response signals toward each EB samples, as we can observe from (**Figure** **3**a). To verify the response signal of standard gas under EB background, spiked EB samples were prepared and tested. Since NH_3_ is one of the commonly reported biomarkers of HP infection^[^ ^20^ ^]^ and a typical trace inorganic gas in EB, the EB samples spiked with 1 ppm NH_3_ were prepared as mentioned above in Section “Analysis of Spiked EB” and measured. The results displayed that the Re% of spiked EB was significantly higher compared to pure EB (Figure 3b; Figure S15, Supporting Information), indicating that the sensor array maintains good sensing sensitivity to the standard gas in the presence of complex EB matrix. Moreover, the 2D and 3D Orthogonal Partial Least Squares‐Discriminant Analysis (OPLS‐DA) and PCA analysis also indicated that the rGO‐PDDA‐M array could effectively distinguish between EB and spiked EB (1 ppm NH_3_) (Figure 3c–f). The results were essential for the application of the sensor array in EB analysis.

### 2.6 Analysis of Clinical EB Samples on the rGO‐PDDA‐M Sensor Array

The gut microbiota is crucial for maintaining a healthy metabolism in the host. This regulation is mediated through the production of various metabolites by the microbiome under both healthy and diseased conditions, along with their underlying molecular mechanisms.^[^ ^2^, ^6^, ^59^ ^]^ Analysis indicates that detecting VOCs in EB can offer valuable insights into the gut microbiota and aid in the diagnosis of various diseases, including gastrointestinal disorders and different types of cancers such as stomach and colorectal cancer.^[^ ^6^, ^60^ ^]^ VOCs are metabolites that are released in the gut, absorbed into the bloodstream, distributed throughout the body, and excreted through respiration via the exchange of gases and blood, which can cause changes in the concentration of VOCs in EB. Breathomics has been a subject of research interest since Linus Pauling identified a complex mixture of ≈250 VOCs in human breath.^[^ ^61^ ^]^ Studies have shown that patients with stomach diseases exhibit significant changes in the levels of alkanes, ketones, and aldehydes in their exhaled breath compared to healthy individuals.^[^ ^8^, ^62^ ^]^ Furthermore, Ulanowska et al. conducted research comparing breath samples from 6 patients with *Helicobacter pylori* and 23 healthy volunteers using GC‐MS, and the results indicated that isobutane, 2‐butanone, and ethyl acetate levels were elevated in the exhaled breath of HP‐positive patients.^[^ ^14b^ ^]^ Meanwhile, another report found that inorganic compounds, such as NO^[^ ^19^ ^]^ and NH_3_,^[^ ^20^ ^]^ were significantly up‐regulated in the exhaled air of *Helicobacter pylori* patients. The above study indicates that breathomics analysis has the potential for non‐invasive, label‐free identification of HP patients.

In this study, the method described above was used to collect and analyze 204 clinical EB samples. The samples consisted of 117 healthy individuals and 87 HP‐positive patients, all of whom were diagnosed using the UBT. The response fingerprints of each sensing layer on the rGO‐PDDA‐M to the 204 clinical samples varied among individuals due to the unique response fingerprints. However, significant differences were observed between the healthy and HP groups (**Figure** **4**a; Figure S16, Supporting Information). The difference in response values may be attributed to variations in the types and contents of gas composition between the two groups of EB samples. Re% and t_50_ in the sensing trace were chosen as characteristic values as input data. Subsequently, OPLS‐DA analysis shows a discernible difference between the healthy group and the HP‐positive patient group (Figure S17, Supporting Information). Nevertheless, there is significant room for improvement in the model's ability to differentiate different groups with R^2^ and Q^2^ of 0.666 and 0.619, respectively. The VIP value, reflecting the variable's contribution to the overall fit and classification ability of the model, was calculated. Variables with higher VIP values are more important in model construction. As shown in Figure S18a (Supporting Information), the VIP value of the Re% variable is higher than that of the t_50_ variable and the contribution of the Re% variable for classification (R^2^ is 0.426; Q^2^ is 0.380) is greater than that of the t_50_ variable (R^2^ is 0.169; Q^2^ is 0.104) when they were analyzed separately (Figures S18b,c, Supporting Information). If the two variables were used together in the OPLS‐DA analysis, the R^2^ and Q^2^ could reach 0.666 and 0.619, respectively (Figure S17, Supporting Information), indicating that the model using the combined variable has a better ability to discriminate EB samples. To further improve discrimination and simplify the sensing elements, the contribution of different composites was further analyzed through the VIP values of the Re% variable (Figure S18a, Supporting Information). The radar map (Figure 4b) showed that the contribution of rGO‐PDDA‐Co, rGO‐PDDA‐Co/Fe, rGO‐PDDA‐Au/Ag, and rGO‐PDDA‐Ce to distinguish between the healthy and HP positive groups was significantly higher than that of the other sites. Thus, the OPLS‐DA analysis was re‐performed based on the Re% and t_50_ variables using only these four sensing layers to eliminate redundant information. We found that the 4‐sensing layer sensor array significantly improved the ability to distinguish between the healthy and HP positive groups with R^2^ and Q^2^ values of 0.827 and 0.759, respectively (Figure 4c). Additionally, the analysis revealed that there were no significant differences between the sexes and age groups in the different EB samples (Figure S19, Supporting Information), further suggesting that the differences in gas response fingerprints were caused by HP infection. Consequently, we employed the 4‐sensing layer array for the subsequent HP diagnosis study.

### 2.7 Construction of Compact Sensor Array

**Figure 5:**
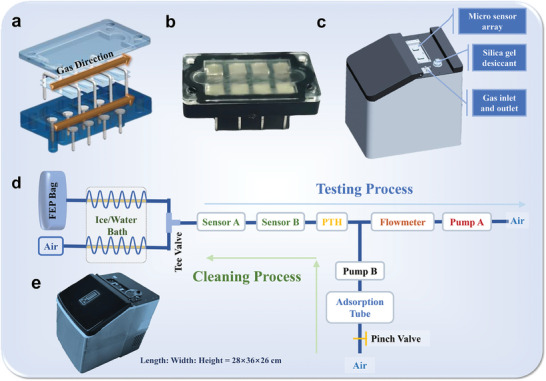
a) The structure diagram of the microsensor array; b) The real picture of the microsensor array; c) The schematic of the e‐nose prototype; d) The working diagram of the e‐nose prototype; e) The photo of the actual e‐nose prototype; The dimensions of the e‐nose prototype were determined to be 22 cm × 36 cm × 26 cm.

A compact sensor array has been designed for potential commercialization. The sensor array can contain up to eight independent chips, which are packed in back‐to‐back pairs (**Figure** **5**a,b). The four composites including rGO‐PDDA‐Co/Fe, rGO‐PDDA‐Au/Ag, rGO‐PDDA‐Ce, and rGO‐PDDA‐Co were selected based on the above analysis results and made into independent sensing chips. The chips were placed face up in the corresponding position on the microsensor array as shown in Figure 5b. Then, conductive glue was used to ensure good contact between the sensor pins and the chips' electrodes. Finally, the pins were pressed and the sensor cover was closed.^[^ ^63^ ^]^

### 2.8 Design of E‐Nose Prototype for Breath Analysis

The e‐nose prototype comprises three modules for achieving the function of gas pre‐treatment, gas detection, and gas path cleaning. The schematic diagram was illustrated in Figure 5c–e. The gas pre‐treatment module is mainly composed of a cooling bath device, which ensures a stable environment temperature and reduces humidity. Specifically, the process of dehumidifying the sample gas (EB) and carrier gas (air) is carried out through the dependent Teflon tubes, during which the humidity levels are equal for both gases, preventing water from affecting the test.

The gas detection module includes a PTH (pressure, temperature, and humidity) sensor, a flow meter, and a micro pump. An electromagnetic valve switches between the air gas (blank)  and sample gas, which are then pumped by a micropump at the end of the flow path. The blue arrow illustrates the gas flow path during the testing process (Figure 5d).

The gas path cleaning module is used to clean the pipelines of the gas pre‐treatment and sensor chip. The cleaning process involves removing the ice water bath atmosphere from the two pre‐treatment pipelines and activating the internal heating wire to heat up to 100 °C, ensuring gas desorption and cleanliness. The green arrow illustrates the gas flow path in the cleaning process (Figure 5d). During the cleaning process, the pinch valve located behind the adsorption tube opens and connects to the air. Air pump B then pumps ambient air through the adsorption tube, which is filled with 4A molecular sieve, to the front part of the gas flow path. The air gas path and sample gas path are cleaned separately by switching the electromagnetic valve.

The non‐equilibrium method was used to conduct the EB test by controlling the response and recovery time. The gas responses Re% and t_50_ were used as characteristics input data to classify the HP‐infected EB sample from healthy individual. After blowing the EB into the sampling bag, the outlet of bag is connected to the inlet of gas path of the e‐nose. Thereafter, the software drove the e‐nose to complete the EB test procedure. The carrier gas and EB were pumped separately by pump A and continuously tested by a compact sensor array to acquire response curves. After testing of each sample, pump A was turned off, and pump B was automatically turned on to introduce dry and clean air at a flow rate of 2L min^−1^ to clean the pipeline.

### 2.9 Establishing the HP Diagnostic Model on the E‐Nose Prototype

**Figure 6:**
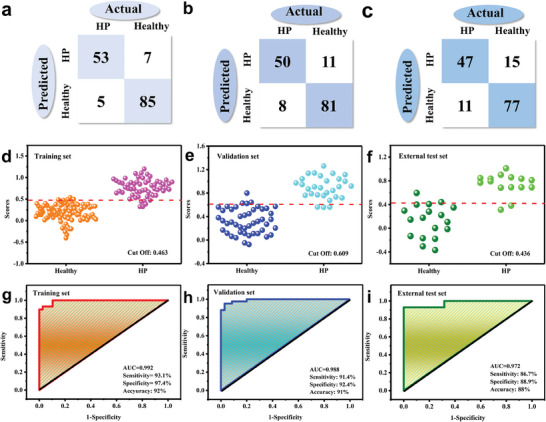
Discriminant results of validation set of EB samples based on Lasso regression model. a) median normalization; b) sum normalization; c) max‐min normalization. Lasso model analysis results of d) training set; e) validation set; and f) external test set of 225 cases EB samples. ROC curves analysis of g) training set; h) validation set; and i) external test set of 225 cases EB samples.

This study involved collecting and analyzing 225 clinical EB samples, including 138 samples from healthy individuals and 87 samples from HP‐positive patients diagnosed using the UBT. The compact sensor array was inserted into the e‐nose prototype device. The response signals of all 225 samples were measured using this equipment (Please refer to Video S1, Supporting Information for the actual test process). Figure S20 (Supporting Information) showed that the sensor sensitivity decreased to ≈25% of its original sensitivity after 30 days, indicating that the sensor had reached the end of its lifespan and required replacement. The entire experiment used two chips of a compact sensor array, in which sensor‐1 tested 101 EB samples and sensor‐2 tested the remaining sample. The representative response curves of healthy individuals and HP‐positive individals on the above 4‐site rGO‐PDDA‐M compact sensor array were shown in Figure S21 (Supporting Information). It can be observed that the responses of the two groups of samples at the 4‐sensing layer array remain significantly different. Considering the differences between the data batches for different days and different sensor tests, acetone gas was employed as the standard gas to calibrate the response data of all EB samples. It can be observed that the response fluctuation of 138 cases of healthy individuals decreased significantly after acetone calibration, and the average inter‐batch RSD% of 4 sensing layers decreased from 32.64% to 9.26% (Figure S22, Supporting Information), proving that acetone is a feasible quality control gas. In the data analysis, three data normalization methods including sum normalization, max‐min normalization and median normalization and two algorithms including Lasso regression and support vector machine (SVM) algorithm were employed, respectively. The 225 EB samples were divided into a training set and a validation set in a 2:1 ratio for Lasso regression modeling analysis. The results indicated that the median normalization method had the highest discrimination in the training set, achieving an accuracy of 92% for HP diagnosis (**Figure** **6**a–c). Furthermore, a SVM model was also constructed using the median normalization method. It yielded an accuracy of 87% (Figure S23, Supporting Information), which was lower than that of the Lasso regression model. Consequently, the Lasso regression method and median normalization method were selected for further analysis.

The compact sensor array with 4‐element rGO‐PDDA‐M exhibited excellent distinguishing performance between healthy and HP groups in both the training and validation sets of the lasso model (Figure 6d,e). The analysis of the receiver operating characteristic (ROC) curves indicated that the area under curve (AUC) values of the training and validation sets were 0.992 and 0.988, respectively (Figure 6g,h). The sensitivity and specificity of the training set were 93.1% and 97.4%, respectively, while those of the validation set were 91.4% and 92.4%, respectively. Overall, the prototype of the e‐nose achieved an accuracy of 92% and 91% in the training and validation sets for HP diagnosis, respectively (Figure 6g,h; Figure S24, Supporting Information). Furthermore, we used 33 cases of EB samples as an external test group to verify the accuracy of the Lasso regression model. 18 cases of healthy individuals and 15 cases of HP‐positive patients were consented and enrolled. The data showed that the model had a good ability to discriminate between healthy and HP‐infected groups with a diagnosis sensitivity and specificity of 86.7% and 88.9%, respectively. Using a cutoff value of 0.436, the overall diagnostic accuracy was 88% with an AUC value of 0.972 (Figure 6f,i; Figure S25, Supporting Information). These above results indicated that the e‐nose has great potential for the label‐free diagnosis of HP infection.

## 3 Conclusion

In summary, a graphene‐based sensor array was constructed by incorporating various metal‐based receptors with PDDA intercalated rGO. The sensor array exhibited high sensitivity and cross‐reactive selectivity to several typical trace gases in EB at room temperature. The sensor array displayed a high discrimination ability toward typical standard gases including NO, NH_3_, isoprene and acetone. After a dehumanization process, EB samples with or without HP infection could be clearly discriminated with a four‐element sensor array. Based on the lab investigation, successful design and construction of a compact sensor array and an engineered e‐nose prototype for HP diagnosis were achieved. Using the prototype of graphene e‐nose, 225 EB samples from people infected with HP and from healthy people were measured and analyzed. The results of the optimized Lasso model indicate that HP‐positive patients can be distinguished from healthy controls with a sensitivity and specificity of 93.1% and 97.4% for the training set, and 91.4% and 92.4% for the validation set, respectively. Additionally, the Lasso regression model was validated in a study of 33 external test cases, achieving 86.7% sensitivity and 88.9% specificity, respectively, with a discrimination accuracy of 88% using a cutoff value of 0.436. The ROC curve analysis showed an AUC value of 0.972. These results display that the e‐nose prototype integrated with the 4‐site rGO‐PDDA‐M compact sensor array has great potential for rapid label‐free HP diagnosis, which may further expand to non‐invasive disease diagnosis.

## 4 Experimental Section

### Reagents and Materials

PET film coated with ITO conductive layer (ITO‐PET) was obtained from South China Science & Technology Co., Ltd (China). Photoresist S1805 (Shipley) for lithography was purchased from Anzhi lithography Electronic Materials Co., Ltd (Germany). Chemical reagents including HCl (36–38%), HNO_3_ (65‐68%), acetone (≥99.5%), and hydrazine hydrate (85%) were purchased from Sinopharm Chemical Reagent Co., Ltd (China). Graphene Oxide (GO) sol was purchased from Chengdu Institute of Organic Chemistry, Chinese Academy of Science (CAS). Poly (diallyl dimethylammonium chloride) (PDDA, Mw. < 10 KDa) and MoS_2_ (≥99%) were purchased from Shanghai Macklin Biochemical Co., Ltd. (China). NaBH_4_ (AR), CuCl_2_ (≥99%), and FeCl_3_·6H_2_O (≥99%), glucose (AR), NH_3_·H_2_O (25%−28%) were purchased from Sinopharm Chemical Reagent Co., Ltd. (China). CoCl_2_·7H_2_O (99.99%) and CeCl_3_·6H_2_O (99.99%), HAuCl_4_·3H_2_O (≥99.9%), AgNO_3_ (AR), were purchased from Aladdin Industrial Co. (China), and molybdenum disulfide (MoS_2_) were purchased from Shanghai Macklin Biochemical Co., Ltd (China). Standard gases including, acetone (102 ppm in Air), NO (10 ppm in N_2_), NO_2_ (10 ppm in Air), NH_3_ (19 ppm in Air), CO (10 ppm in N_2_), H_2_S (9.8 ppm in Air), isoprene (504 ppm in N_2_), high purity air, and N_2_ were purchased from Hangzhou Jingong Special Gas Co., Ltd, China.

### Preparation of GO‐PDDA‐M Composites and Sensor Array Chip

The 2D‐MoS_2_ and GO solutions were obtained by the ultrasonic peeling method as described in the previous work.^[^ ^33^ ^]^

AuNPs were synthesized by the traditional method with HAuCl_4_·3H_2_O and NaBH_4_. Briefly, 200 µL freshly prepared NaBH_4_ (0.1 m) was added to 5 mL HAuCl_4_ (10 mm) under stirring in an ice water bath. After a 30 min reaction, the wine‐red AuNPs solution was obtained. AgNPs were prepared with reference to the reported method.^[^ ^64^ ^]^

rGO‐PDDA‐M composites were obtained by the polymer‐assisted liquid phase self‐assembly method.^[^ ^33^ ^]^ In a typical procedure, 15 µL (0.015 vol%) of PDDA (Mw. < 10 KDa, 1% Wt) was added to 0.5 mL GO (0.5 mg mL^−1^), and then 300 µL freshly prepared NaBH_4_ (0.1 m) was added to the above solution under stirring for 10 min. The method of post‐treatment of the suspension was the same as in the previous work.^[^ ^33^ ^]^ Then, 0.5 mL CoCl_2_ (15 mm) was added to 0.5 mL rGO‐PDDA. The suspension underwent vibration at room temperature on an oscillator for a duration of 16 h with a shaking speed of 1000 rpm. Subsequently, the suspension was post‐treatment as described above. Other metal‐based rGO‐PDDA‐M composites were synthesized in the same way, except that the concentration of Fe^3+^ and Ce^3+^ was 10 mm and the concentration of Cu^2+^ was 20 mm. The AuNPs and AgNPs were prepared as described above. The rGO was prepared using a similar procedure as described above, with the exception of the addition of PDDA and metal‐based materials.

The preparation process of the 8‐channel IDEs and the sensor chip was described in detail in the previous work.^[^ ^33^ ^]^

### Materials Characterization

The morphologies of rGO‐PDDA‐M composites were observed using scanning electron microscope (SEM, Hitachi, SU8010, Japan). The elemental analysis was conducted via X‐ray photoelectron spectroscopy (XPS) using a VG ESCALAB MKII system from the UK, equipped with an Mg QR X‐ray radiation source. Raman spectra of the rGO‐PDDA‐M films were characterized using a Jobin Yvon Labor Raman spectrometer (HR‐800, France) with a 514.5 nm laser.

### Measurement of Gas Sensor Array's Response—Standard Gas Sensing

The gas concentration was adjusted as described in previous work by the research group.^[^ ^33^ ^]^ The sensor's resistance was read out and recorded by a multi‐channel measure instrument (Will‐think Sensing Technology Co., China) and La IEW 8.6 software, respectively. The sensor array was tested using a measurement system manufactured in the laboratory, and the tested resistance was subsequently transformed into the relative response signal (Re%) as follows:

(1) $$Re\%\;=\frac{R-R_{0}}{R_{0}}\;\times 100$$

Here, *R*_0_ represents the resistance measured in the stream of carrier gas, and *R* is the resistance when exposed to analytes. The half of recovery time (t_50_) refers to the duration required for the resistance to restore to 50% of its initial resistance value.

### Measurement of Gas Sensor Array's Response—EB Analysis

*EB Sample Collection and Measurement Method*: The collection and testing methods of EB samples were detailed in the previous work.^[^ ^33^, ^34^ ^]^

To mitigate the impact of humidity in EB analysis, a condensation method was employed. Briefly, a nine‐grid ice pack was packed in insulated paper into a cylinder shape and then pass a Teflon tube through the cylindrical condensing pipe, through which the EB sample gas flows. This process effectively reduces and stabilizes the humidity of the collected gas.

*Analysis of Spiked EB*: To prepare spiked EB samples for analysis, standard NH_3_ was added to EB samples collected from 10 healthy individuals. In brief, 10.5 mL of 19 ppm NH_3_ was injected into an FEP bag containing the EB sample of healthy people to achieve a final NH_3_ concentration of 1 ppm. For the control, an identical volume of N_2_ was introduced into a separate FEP bag containing the same EB sample. Both bags were left to stabilize at room temperature for 2 h. These prepared samples were labeled as NH_3_ (Blank) and NH_3_ (1 ppm).

*Analysis of Clinical EB*: A total of 462 volunteers from the Second Affiliated Hospital of Zhejiang University School of Medicine participated in the study, of which 204 cases were tested on a simple cooling device and data acquisition module made in the laboratory, and the remaining 258 EB samples were analyzed on a prototype machine designed and developed later. For clinical EB analysis, to minimize batch‐to‐batch data variation, the original relative response signals (Re%) on each sensor layer underwent calibration using a standard acetone gas as a reference. And this study was conducted from April 20, 2022 to November 30, 2023. The entire collection of samples was obtained from 28 batches, with each batch comprising a range of 14 to 18 samples. These samples were then tested in a random order within the day of their collection. The proportion of healthy and patient samples was consistent with the incidence rate. Informed consent was obtained from each patient and monitored by the Ethics Review Board of the Second Affiliated Hospital of Zhejiang University of Medicine (Ethical Review No. I2023074) (Figure S26, Supporting Information).

*Statistical Analysis*: In the analysis of standard gas and EB samples, the data were preprocessed by sum normalization and median normalization, respectively. The response data was represented by the mean ± SD. Moreover, in the preliminary analysis of the HP samples in Section 2.6, the sample size (n) was 204. In the analysis of the HP samples in Section 2.9 on the prototype sensor, the sample size (n) was 258, including 33 external test sets. The Mann‐Whitney U test was employed for significance difference analysis, and two‐sided testing was used. When the alpha value was 0.05 and the *P* < 0.05, the two groups of samples were considered to have significant differences. Significance difference analysis was performed using IBM SPSS 26.0. In addition, modeling analysis was performed using MATLAB R2019a, and ten‐fold cross‐validation was used for both Lasso and SVM analysis.

## Conflict of Interest

The authors declare no conflict of interest.

## Supporting information

Supporting Information

## Data Availability

The data that support the findings of this study are available from the corresponding author upon reasonable request.

## References

[1] a) J. R. Warren , Lancet 1983, 4, 1273; 6134060

[2] a) A. L. Dinca , L. E. Melit , C. O. Marginean , Children (Basel) 2022, 9, 1083. 35884067 10.3390/children9071083 PMC9322908

[3] a) J. K. Y. Hooi , W. Y. Lai , W. K. Ng , M. M. Y. Suen , F. E. Underwood , D. Tanyingoh , P. Malfertheiner , D. Y. Graham , V. W. S. Wong , J. C. Y. Wu , F. K. L. Chan , J. J. Y. Sung , G. G. Kaplan , S. C. Ng , Gastroenterology 2017, 153, 420; 28456631 10.1053/j.gastro.2017.04.022

[4] a) P. Malfertheiner , F. Megraud , C. A. O'Morain , J. Atherton , A. T. Axon , F. Bazzoli , G. F. Gensini , J. P. Gisbert , D. Y. Graham , T. Rokkas , E. M. El‐Omar , E. J. Kuipers , Gut 2012, 61, 646;22491499

[5] a) D. Liu , R. Zhang , S. Chen , B. Sun , K. Zhang , BMC Microbiol. 2022, 22, 184; 35870901 10.1186/s12866-022-02594-y PMC9308235

[6] P. Malfertheiner , M. C. Camargo , E. El‐Omar , J. M. Liou , R. Peek , C. Schulz , S. I. Smith , S. Suerbaum , Nat. Rev. Dis. Primers 2023, 9, 19. 37081005 10.1038/s41572-023-00431-8 PMC11558793

[7] a) J. Baj , A. Forma , W. Flieger , I. Morawska , A. Michalski , G. Buszewicz , E. Sitarz , P. Portincasa , G. Garruti , M. Flieger , G. Teresinski , Cells 2021, 10, 2191. 34571840 10.3390/cells10092191 PMC8469861

[8] J. Chung , S. Akter , S. Han , Y. Shin , T. G. Choi , I. Kang , S. S. Kim , Int. J. Mol. Sci. 2022, 24, 129. 36613569 10.3390/ijms24010129 PMC9820758

[9] G. Y. Pih , J. H. Noh , J. Y. Ahn , G. S. Han , H. S. Jung , H. Y. Jung , J. M. Kim , J. Korean Med. Sci. 2022, 37, 227. 10.3346/jkms.2022.37.e227 PMC9313973 35880503

[10] K. G. Hulten , R. M. Genta , I. N. Kalfus , Y. Zhou , H. Zhang , D. Y. Graham , Gastroenterology 2021, 161, 1433. 34293298 10.1053/j.gastro.2021.07.012 PMC9047521

[11] V. Weingart , H. Russmann , S. Koletzko , J. Weingart , W. Hochter , M. Sackmann , J. Clin. Microbiol. 2004, 42, 1319. 15004108 10.1128/JCM.42.3.1319-1321.2004 PMC356865

[12] X. Wang , S. Zhang , E. G. Chua , Y. He , X. Li , A. Liu , H. Chen , M. J. Wise , B. J. Marshall , D. Sun , X. Li , C. Y. Tay , Gut Pathog. 2021, 13, 38. 34118962 10.1186/s13099-021-00435-3 PMC8199820

[13] A. Maity , G. D. Banik , C. Ghosh , S. Som , S. Chaudhuri , S. B. Daschakraborty , S. Ghosh , B. Ghosh , A. K. Raychaudhuri , M. Pradhan , J. Breath Res. 2014, 8, 016005. 24566134 10.1088/1752-7155/8/1/016005

[14] a) J. A. Ferreira , E. Dias , S. M. Rocha , M. A. Coimbra , Anal. Bioanal. Chem. 2011, 401, 1889; 21822779 10.1007/s00216-011-5259-x

[15] A. A. Oraevsky , H. Harde , L. V. Wang , G. Helmrich , M. Wolff , presented at Photons Plus Ultrasound: Imaging and Sensing , San Francisco, California, U.S. 2010.

[16] I. Bayrakli , A. Turkmen , M. Cem Kockar , Appl. Spectrosc. 2016, 70, 1269. 27296306 10.1177/0003702816654052

[17] a) T. H. Risby , S. F. Solga , Appl. Phys. B 2006, 85, 421;

[18] T. L. Mathew , P. Pownraj , S. Abdulla , B. Pullithadathil , Diagnostics (Basel) 2015, 5, 27. 26854142 10.3390/diagnostics5010027 PMC4665550

[19] S. Som , G. Dutta Banik , A. Maity , S. Chaudhuri , M. Pradhan , J. Breath Res. 2018, 12, 026005. 28947681 10.1088/1752-7163/aa8efb

[20] H. Y. Li , C. S. Lee , D. H. Kim , J. H. Lee , ACS Appl. Mater. Interfaces 2018, 10, 27858. 30051712 10.1021/acsami.8b09169

[21] J. Kasperski , M. Wyszynskai , S. Kustra , E. Czecior , M. Misiolek , A. Kasperska‐Zajac , Eur. J. Inflamm. 2013, 11, 279.

[22] Z. Q. Xu , Y. Y. Broza , R. Ionsecu , U. Tisch , L. Ding , H. Liu , Q. Song , Y. Y. Pan , F. X. Xiong , K. S. Gu , G. P. Sun , Z. D. Chen , M. Leja , H. Haick , Br. J. Cancer 2013, 108, 941. 23462808 10.1038/bjc.2013.44 PMC3590679

[23] H. Amal , M. Leja , Y. Y. Broza , U. Tisch , K. Funka , I. Liepniece‐Karele , R. Skapars , Z. Q. Xu , H. Liu , H. Haick , J. Breath Res. 2013, 7, 047102. 24184568 10.1088/1752-7155/7/4/047102

[24] Z. Yildirim , B. Bozkurt , D. Ozol , F. Armutcu , R. Akgedik , H. Karamanli , D. Kizilirmak , M. Ikizek , Helicobacter 2016, 21, 389. 27061444 10.1111/hel.12302

[25] A. M. Diskin , P. Spanˇel , D. Smith , Physiological measurement, 2003, 24, 107. 12636190 10.1088/0967-3334/24/1/308

[26] L. Shi , H. Wang , X. Wu , D. Wang , Q. Zhang , B. Han , J. Sun , X. Wei , C. Li , J. Micromech. Microeng. 2022, 32, 014002.

[27] J. Qian , F. Tian , Y. Luo , M. Lu , A. Zhang , IEEE Trans. Indus. Electron. 2022, 69, 5314.

[28] J. H. Cha , D. H. Kim , S. J. Choi , W. T. Koo , I. D. Kim , Anal. Chem. 2018, 90, 8769. 29790330 10.1021/acs.analchem.8b01273

[29] Q. Li , D. Chen , J. Miao , S. Lin , Z. Yu , Y. Han , Z. Yang , X. Zhi , D. Cui , Z. An , ACS Appl. Mater. Interfaces 2020, 12, 25243. 32391684 10.1021/acsami.9b22098

[30] a) R. Kalidoss , S. Umapathy , R. Anandan , V. Ganesh , Y. Sivalingam , Anal. Chem. 2019, 91, 5116; 30869871 10.1021/acs.analchem.8b05670

[31] a) Y. Chen , F. Meng , M. Li , J. Liu , Sens. Actuators, B 2009, 140, 396;

[32] a) A. Chen , R. Liu , X. Peng , Q. Chen , J. Wu , ACS Appl. Mater. Interfaces 2017, 9, 37191; 28910069 10.1021/acsami.7b11244

[33] X. Liu , Z. He , S. Xu , J. Wu , J. Wu , Sens. Actuators, B 2022, 367, 132185.

[34] Q. Chen , Z. Chen , D. Liu , Z. He , J. Wu , ACS Appl. Mater. Interfaces 2020, 12, 17713. 32203649 10.1021/acsami.0c00720

[35] Q. Wang , R. Li , P. Wang , Y. Zhang , Y. Wang , Y. Yang , Z. Wu , B. An , J. Li , E. Xie , Sens. Actuators, B 2023, 390, 133985.

[36] a) N. Raghuwanshi , P. Kumari , A. K. Srivastava , P. Vashisth , T. C. Yadav , R. Prasad , V. Pruthi , Mater. Sci. Eng. C Mater. Biol. Appl. 2017, 80, 252; 28866163 10.1016/j.msec.2017.05.134

[37] S. Y. Wang , D. S. Yu , L. M. Dai , D. W. Chang , J. B. Baek , ACS Nano 2011, 5, 6202. 21780760 10.1021/nn200879h

[38] J.‐L. Fan , X.‐F. Hu , W.‐W. Qin , M. Zhou , Y.‐S. Liu , S. Cheng , S.‐J. Gao , L.‐P. Tan , G.‐Q. Wang , W. Zhang , J. Mater. Chem. C 2023, 11, 2364.

[39] L. Du , Y. Liu , X. You , H. Sun , Sens. Actuators, B 2024, 401, 135021.

[40] a) X. Wang , R. Wang , Q. Kang , L. Yan , T. Ma , D. Li , Y. Xu , H. Ge , Colloids Surf. A 2023, 656, 130325;

[41] L. Zhang , Q. Fang , Y. Huang , K. Xu , P. K. Chu , F. Ma , Anal. Chem. 2018, 90, 9821. 30033722 10.1021/acs.analchem.8b01768

[42] Q. Hu , B. Huang , Y. Li , S. Zhang , Y. Zhang , X. Hua , G. Liu , B. Li , J. Zhou , E. Xie , Z. Zhang , Sens. Actuators, B 2020, 307, 127638.

[43] X. Cui , X. Tian , X. Xiao , T. Chen , Y. Wang , Adv. Mater. Technol. 2023, 8, 2300572.

[44] S. Xu , X. Liu , J. Wu , J. Wu , ACS Sens. 2023, 8, 2348. 37312238 10.1021/acssensors.3c00428

[45] S. Singh , I. S. Saggu , K. Chen , Z. Xuan , M. T. Swihart , S. Sharma , ACS Appl. Mater. Interfaces 2022, 14, 40382. 36001381 10.1021/acsami.2c09069

[46] T. He , S. Sun , B. Huang , X. Li , ACS Appl. Mater. Interfaces 2023, 15, 4194. 36631735 10.1021/acsami.2c18097

[47] S. Brahma , P. C. Huang , B. W. Mwakikunga , V. Saasa , A. A. Akande , J.‐L. Huang , C.‐P. Liu , Mater. Chem. Phys. 2023, 294, 127053.

[48] K. K. Bedala , P. Gonugunta , M. Soleimani , E. Mádai , P. Taheri , S. K. Padamati , P. Nagaraju , G. Upender , B. Vijaya Kumar , Appl. Surf. Sci. 2023, 640, 158315.

[49] B. Sun , H. Lv , Z. Liu , J. Wang , X. Bai , Y. Zhang , J. Chen , K. Kan , K. Shi , J. Mater. Chem. A 2021, 9, 6335.

[50] A. Yadav , A. Sharma , V. Baloria , P. Singh , G. Gupta , Ceram. Int. 2023, 49, 7853.

[51] A. Sharma , U. Varshney , A. Yadav , P. Vashishtha , P. Singh , G. Gupta , Mater. Chem. Phys. 2023, 296, 127241.

[52] a) L. Yan , Y. Xu , P. Chen , S. Zhang , H. Jiang , L. Yang , Y. Wang , L. Zhang , J. Shen , X. Zhao , L. Wang , Adv. Mater. 2020, 32, 2003313; 10.1002/adma.202003313 33073399

[53] E. Dovgolevsky , G. Konvalina , U. Tisch , H. Haick , J. Phys. Chem. C 2010, 114, 14042.

[54] a) J. Lee , E. Hwang , E. Lee , S. Seo , H. Lee , Chemistry 2012, 18, 5155; 22434768 10.1002/chem.201103554

[55] Z. Bo , X. Guo , X. Wei , H. Yang , J. Yan , K. Cen , Phys. E 2019, 109, 156.

[56] M. Ali , N. Tit , Surf. Sci. 2019, 684, 28.

[57] a) S. S. Yu , T. H. Lee , T. H. Oh , Fuel 2022, 315, 123151;

[58] a) T. Kim , Q. Zhang , J. Li , L. Zhang , J. V. Jokerst , ACS Nano 2018, 12, 5615; 29746090 10.1021/acsnano.8b01362 PMC8045556

[59] W. M. de Vos , H. Tilg , M. Van Hul , P. D. Cani , Gut 2022, 71, 1020. 35105664 10.1136/gutjnl-2021-326789 PMC8995832

[60] a) A. M. Thomas , P. Manghi , F. Asnicar , E. Pasolli , F. Armanini , M. Zolfo , F. Beghini , S. Manara , N. Karcher , C. Pozzi , S. Gandini , D. Serrano , S. Tarallo , A. Francavilla , G. Gallo , M. Trompetto , G. Ferrero , S. Mizutani , H. Shiroma , S. Shiba , T. Shibata , S. Yachida , T. Yamada , J. Wirbel , P. Schrotz‐King , C. M. Ulrich , H. Brenner , M. Arumugam , P. Bork , G. Zeller , et al., Nat. Med. 2019, 25, 667; 30936548 10.1038/s41591-019-0405-7 PMC9533319

[61] L. Pauling , A. B. Robinson , R. Teranishi , P. Cary , Acad. Sci. 1971, 68, 2374. 10.1073/pnas.68.10.2374 PMC389425 5289873

[62] M. P. Bhandari , I. Polaka , R. Vangravs , L. Mezmale , V. Veliks , A. Kirshners , P. Mochalski , E. Dias‐Neto , M. Leja , Molecules 2023, 28, 3488. 37110724 10.3390/molecules28083488 PMC10141340

[63] J. M. Wu , Q. F. Chen , H. Ma , X. H. Chen , W. J. Zhang , X. G. Ye , (Will‐think Sensing Technology Co., LTD, Hangzhou), ZL 2022 1 0480848.3, 2024.

[64] Y. Cui , W. Duan , Y. Jin , F. Wo , F. Xi , J. Wu , ACS Sens. 2020, 5, 2096. 32450686 10.1021/acssensors.0c00718

