# Supplementary material for: A Prototype of Graphene E‐Nose for Exhaled Breath Detection and Label‐Free Diagnosis of Helicobacter Pylori Infection
Source: Adv Sci (Weinh). 2024 Jul 4;11(34):2401695. doi: 10.1002/advs.202401695 (PMC11425842; doi:10.1002/advs.202401695)
Supplement: Supplementary file 1 — Supporting Information [file ADVS-11-2401695-s001.docx]

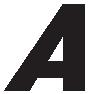

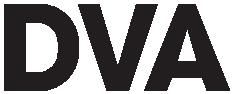

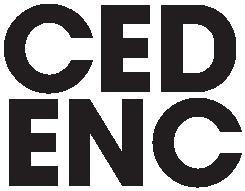

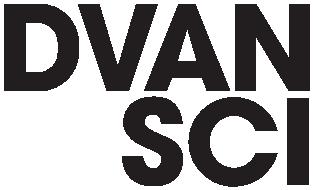

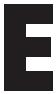


Supporting Information


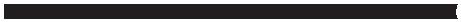

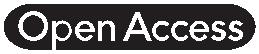


A Prototype of Graphene E-nose for Exhaled Breath Detection and Label-free Diagnosis of *Helicobacter Pylori* Infection

*Xuemei Liu*†*, Qiaofen Chen*†*, Shiyuan Xu, Jiaying wu, Jingwen Zhao, Zhengfu He*, Aiwu Pan*, and Jianmin Wu**


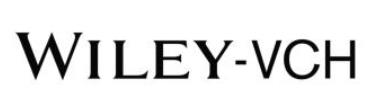
Supporting Information

**A Prototype of Graphene E-nose for Exhaled Breath Detection and Label-free Diagnosis of *Helicobacter Pylori* Infection**

*Xuemei Liu*†*, Qiaofen Chen*†*, Shiyuan Xu, Jiaying wu, Jingwen Zhao, Zhengfu He*, Aiwu Pan*, and Jianmin Wu**

X.M. Liu†, Q.F. Chen†, S.Y. Xu, J.Y. Wu, J.W. Zhao, J.M. Wu

Lab of Nanomedicine and Omic-based Diagnostics, Institute of Analytical Chemistry, Department of Chemistry, Zhejiang University, Hangzhou, 310058, China.

E-mail: [wjm-st1@zju.edu.cn](mailto:wjm-st1@zju.edu.cn)

A.W. Pan

Department of Internal Medicine, The Second Affiliated Hospital of Zhejiang University, Hangzhou, 310003, China.

Email: awpan818@hotmail.com

Z.F He

Department of Thoracic Surgery, Sir Run Run Shaw Hospital, School of Medicine, Zhejiang University, Hangzhou, 310016, China.

Email: [hezhengfu123@hotmail.com](mailto:hezhengfu123@hotmail.com)

Q.F. Chen

Will-think Sensing Technology Co., LTD, Hangzhou, 310030, China.

Email: chenqiaofen@well-healthcare.com


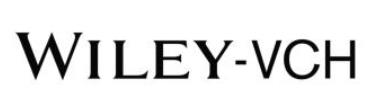


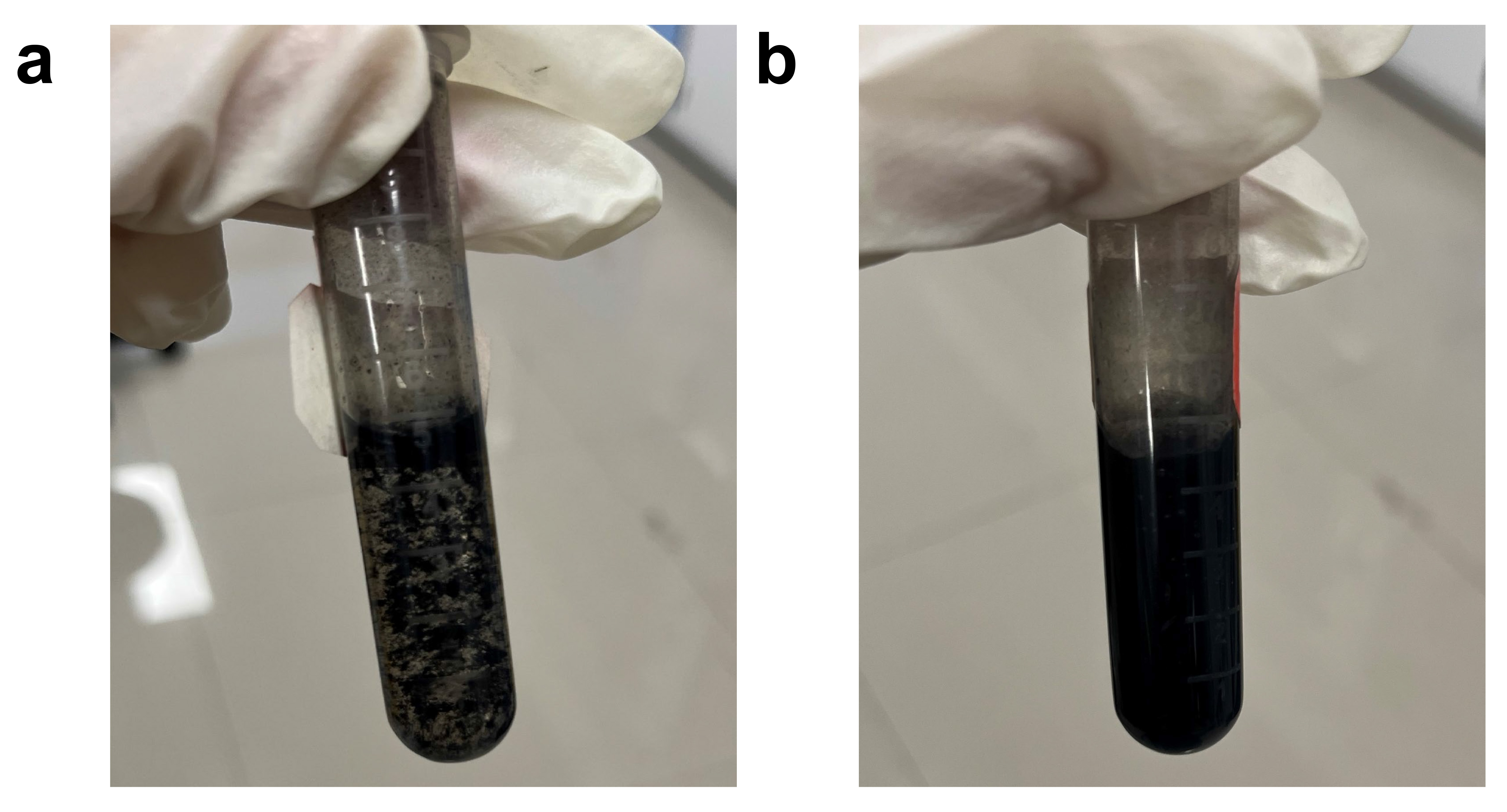


**Figure S1.** Images of liquid phase reduction of rGO in the (a) presence and (b) absence of PDDA


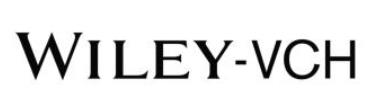


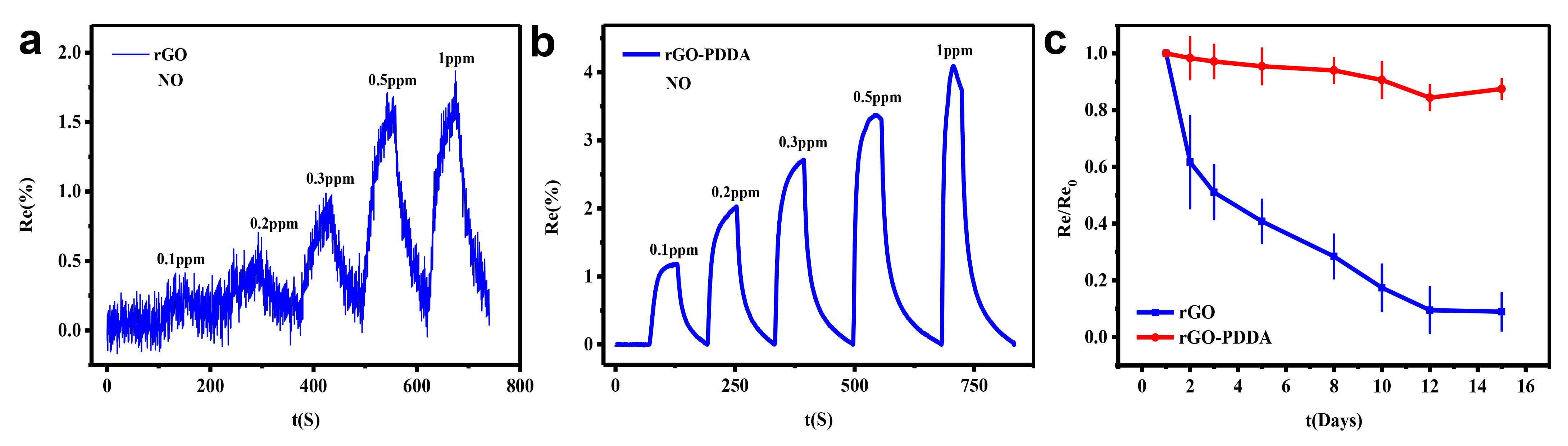


**Figure S2.** (a) Response curves of (a) rGO and (b) rGO-PDDA of liquid phase reduction toward NO gas; (c) Response decay (Re/Re_0_) of rGO and rGO-PDDA toward NO gas within 15 days; Re is the relative response of sensor at different times, whereas Re_0_ is the initial relative response of the sensor.


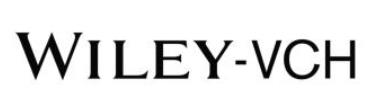


**
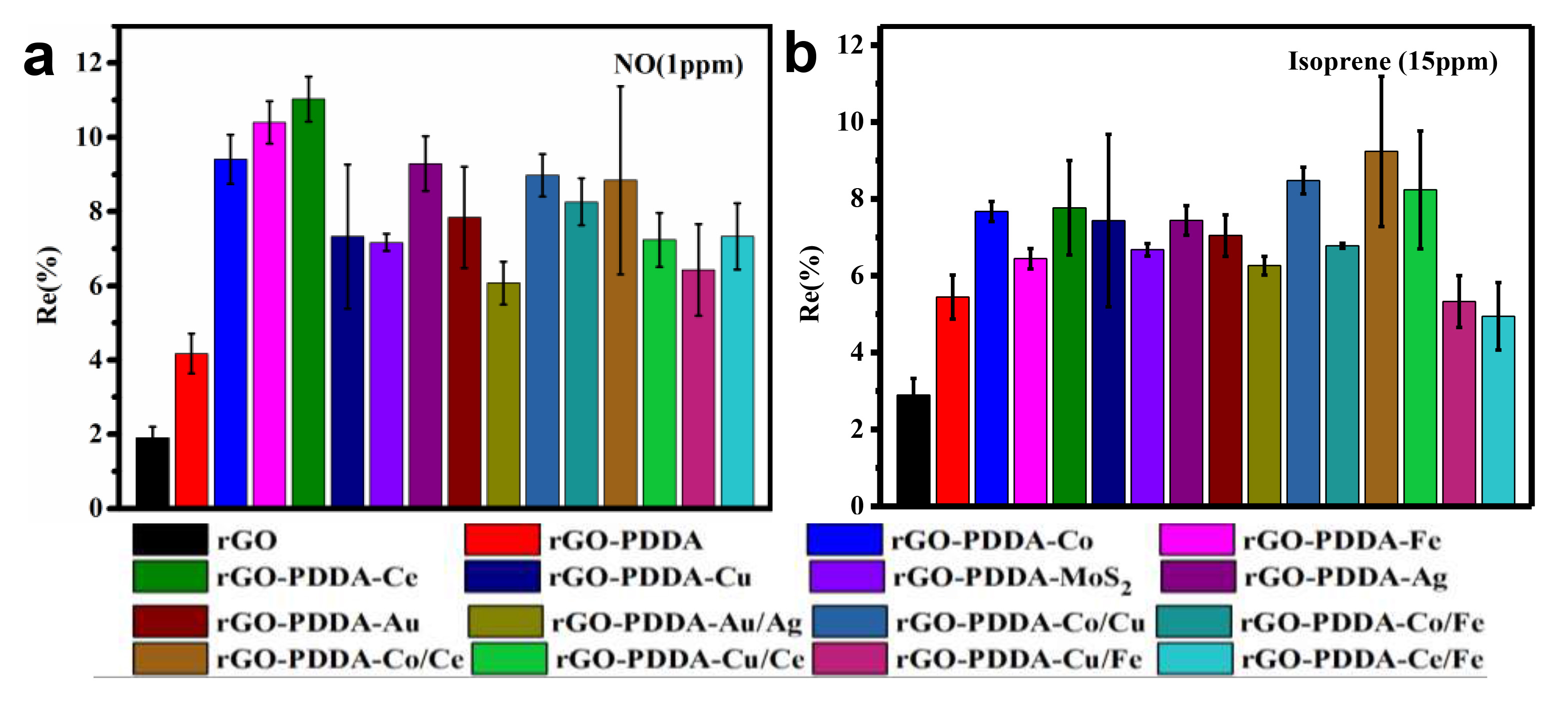
**

**Figure S3.** Response values of different sensing materials toward (a) 1ppm NO; (b) 15ppm isoprene.


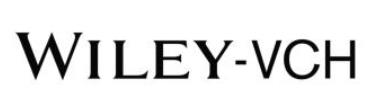


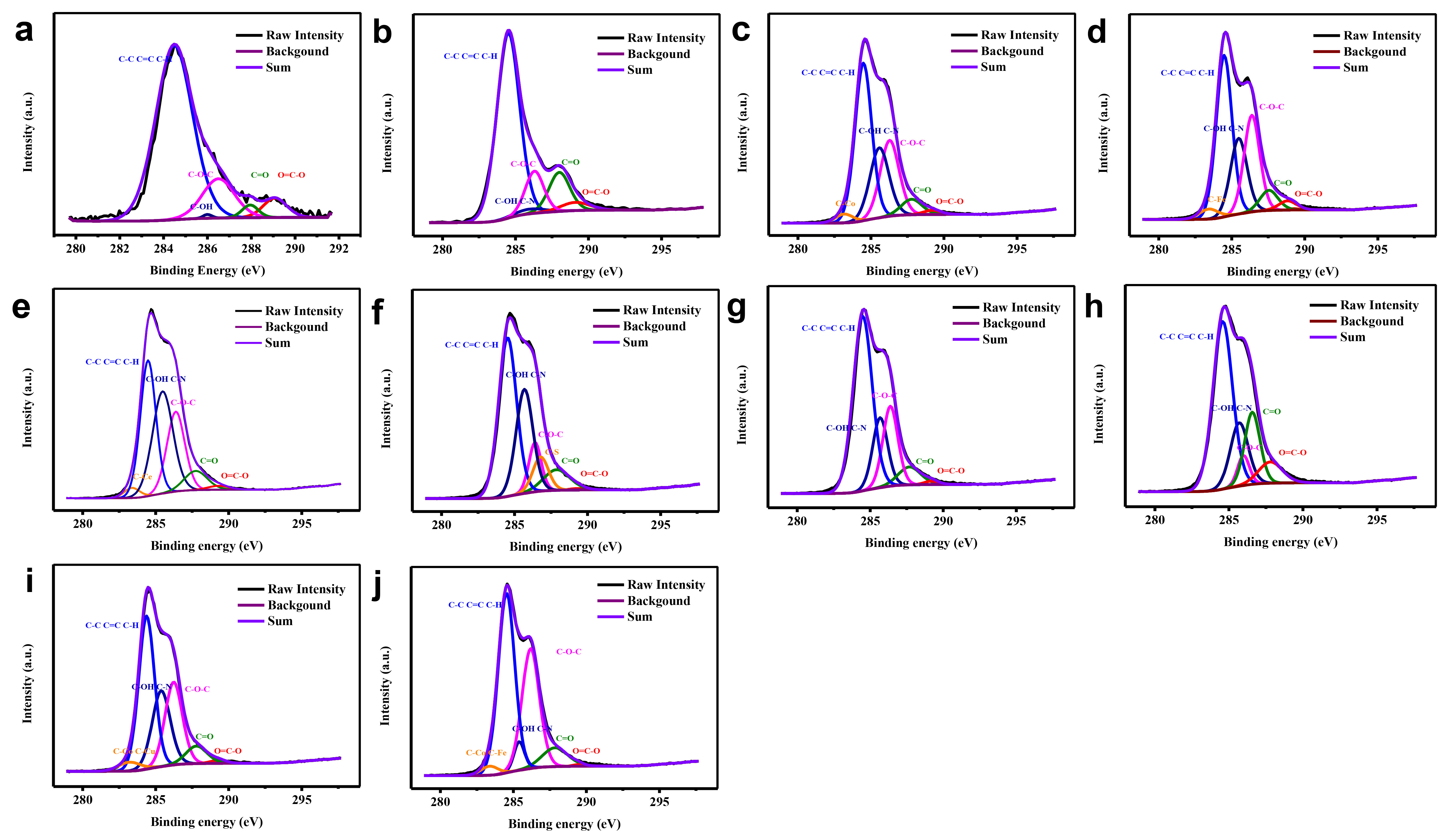


**Figure S4.** The XPS high-resolution C1S scan spectra and peak fitting results of different materials. (a) rGO; (b) rGO-PDDA; (c) rGO-PDDA-Co; (d) rGO-PDDA-Fe; (e) rGO-PDDA-Ce; (f) rGO-PDDA-MoS_2_; (g) rGO-PDDA-Ag; (h) rGO-PDDA-Au/Ag; (i) rGO-PDDA-Co/Cu; (j) rGO-PDDA-Co/Fe.


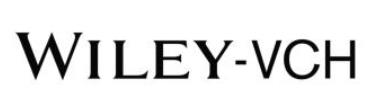


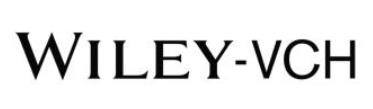


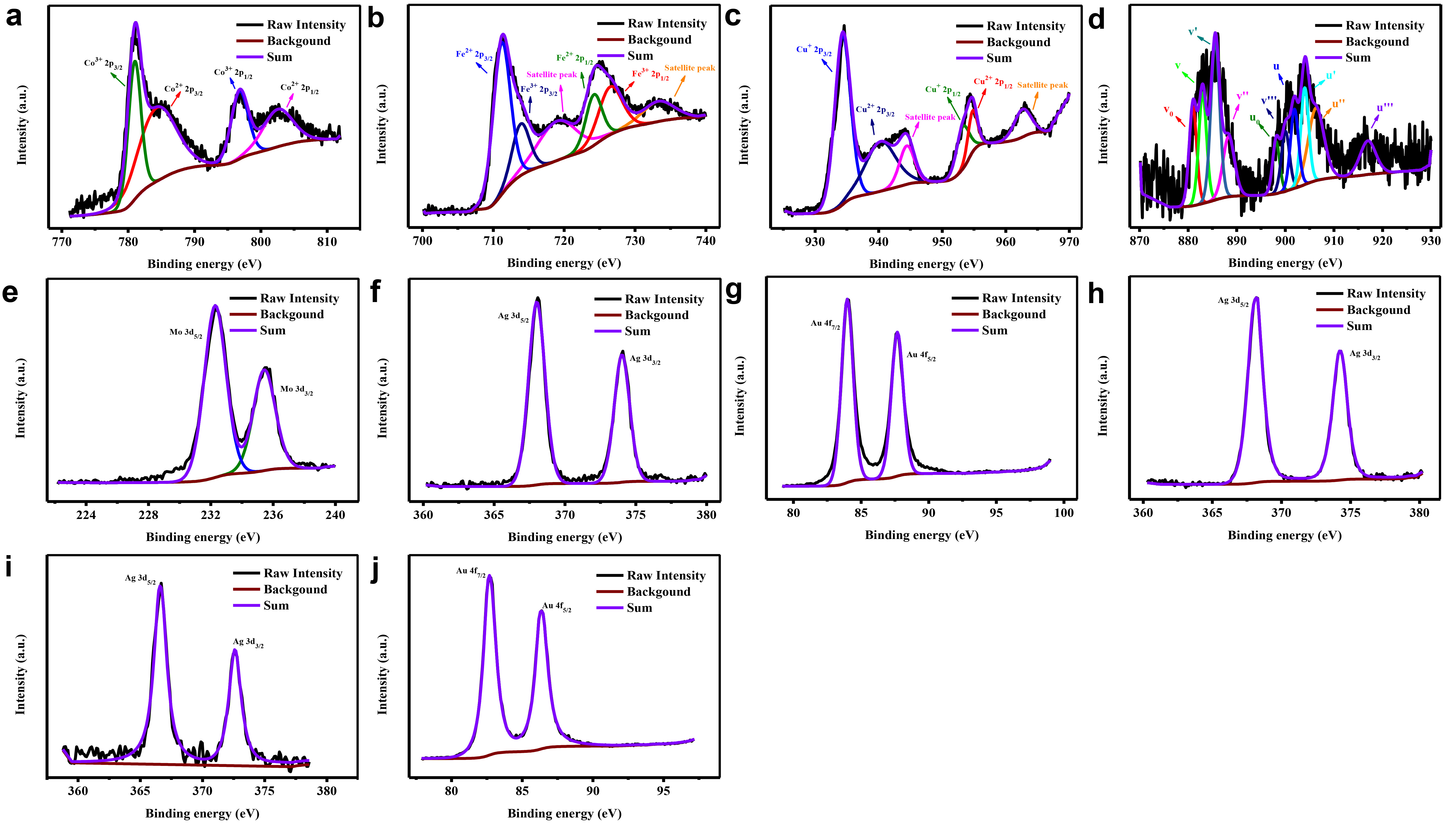


**Figure S5.** The XPS high-resolution scan spectra and peak fitting results of different materials. (a) Co 2p region of rGO-PDDA-Co; (b) Fe 2p region of rGO-PDDA-Fe; (c) Cu 2p region of rGO-PDDA-Co/Cu; (d) Ce 3d region of rGO-PDDA-Ce; (e) Mo 3d region of rGO-PDDA- MoS_2_; (f) Ag 3d region of rGO-PDDA-Ag; (g) Au 4f region of rGO-PDDA-Au/Ag; (h) Ag 3d region of rGO-PDDA-Au/Ag; (i) Ag 3d region of pure AgNPs; (j) Au 4f region of pure AuNPs.


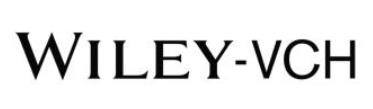

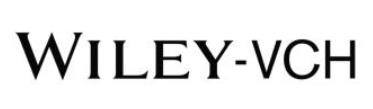


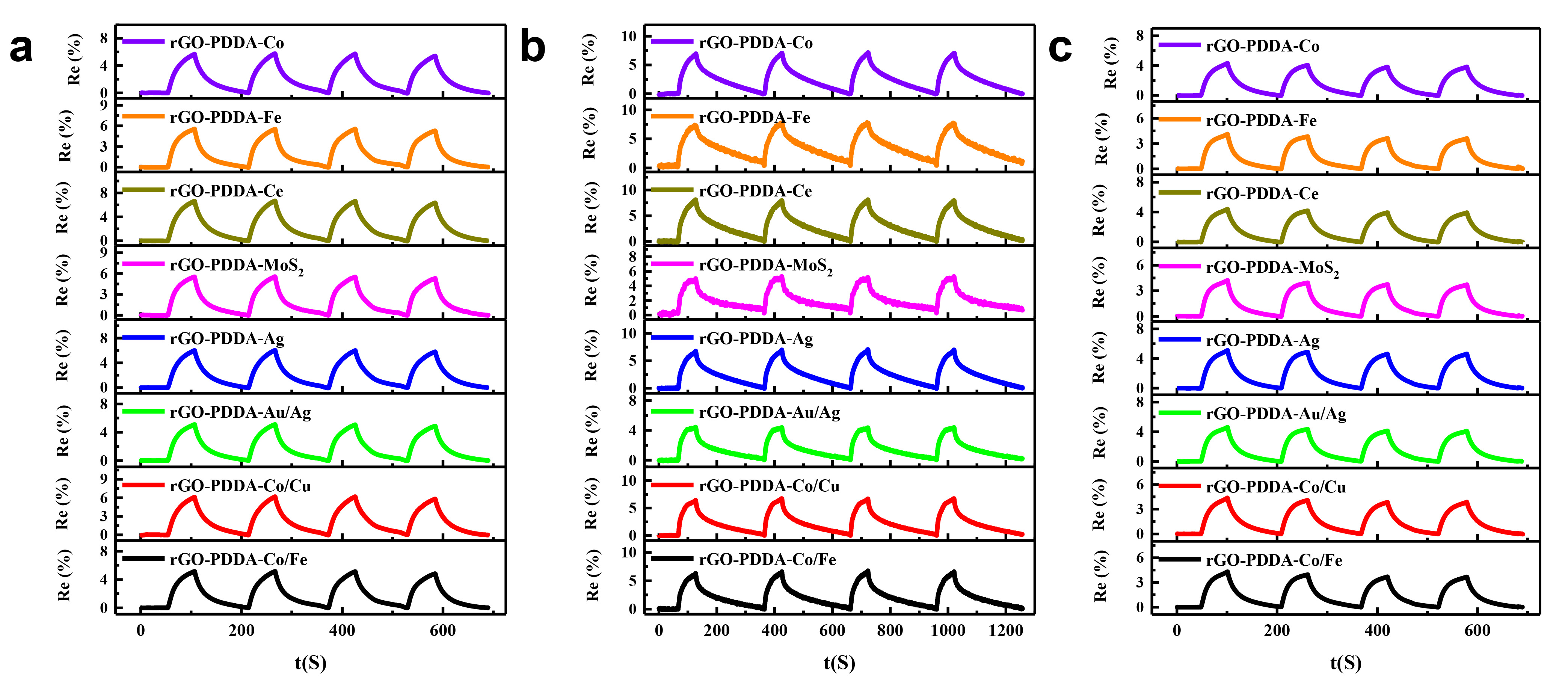


**Figure S6.** Response repeatability of rGO-PDDA-M array to (a) 5 ppm Acetone; (b) 0.5 ppm NO; (c) 0.5 ppm NH_3_.


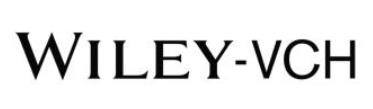

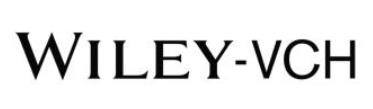


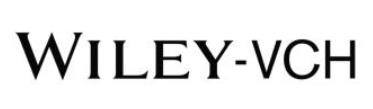


**
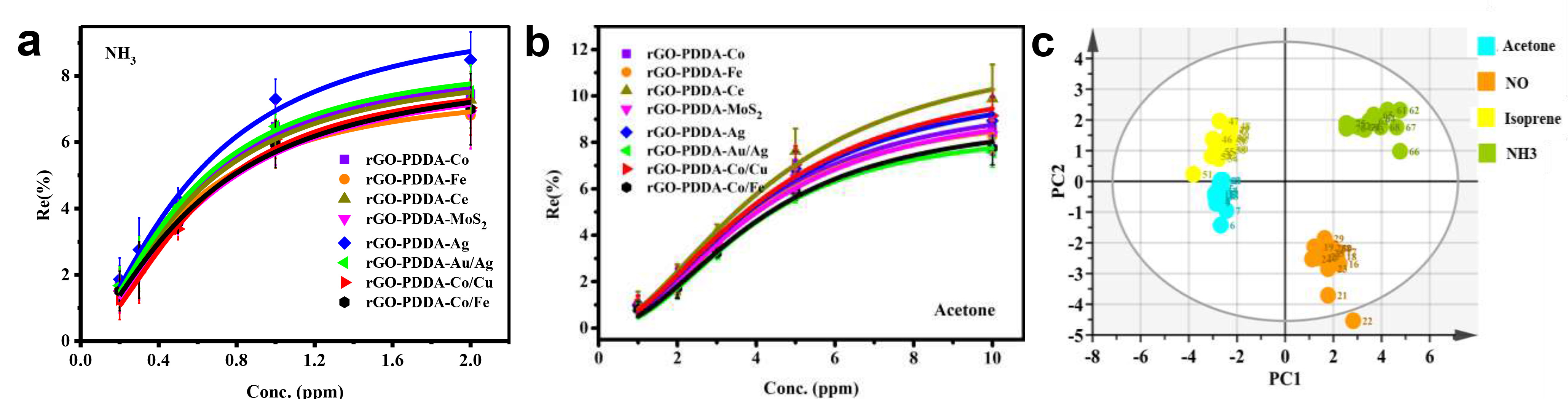
**

**Figure S7.** Langmuir−Hill fitting curves of rGO-PDDA-M sensor array in (a) NH_3_; (b) acetone. (c) PCA result according to normalized Re% of acetone (1−10 ppm), isoprene (2−15 ppm), NO (0.1−1 ppm), and NH_3_ (0.2−2 ppm).


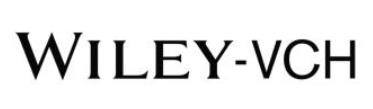


**
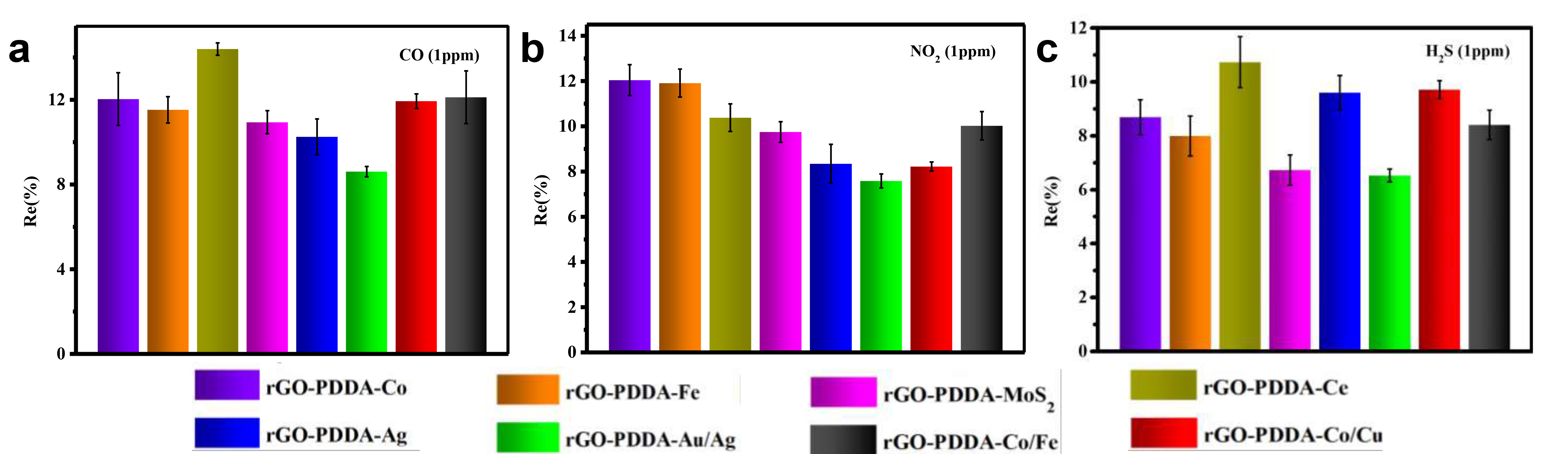
**

**Figure S8.** Response values of rGO-PDDA-M composites toward (a) CO; (b) NO_2_; (c) H_2_S.


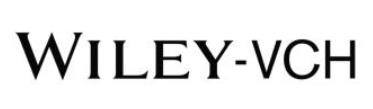


**
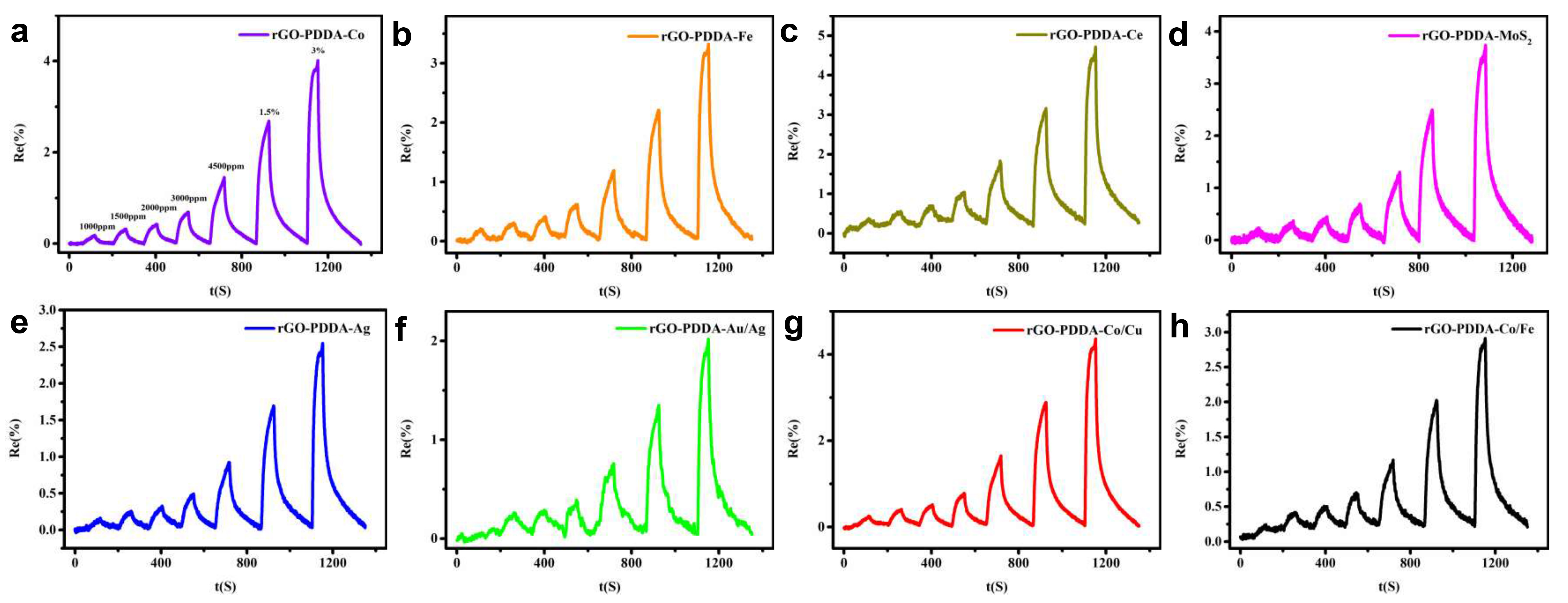
**

**Figure S9.** Response dynamic curves of rGO-PDDA-M sensors array toward different concentrations of CO_2_.


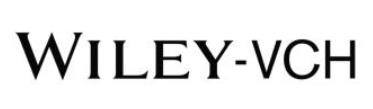


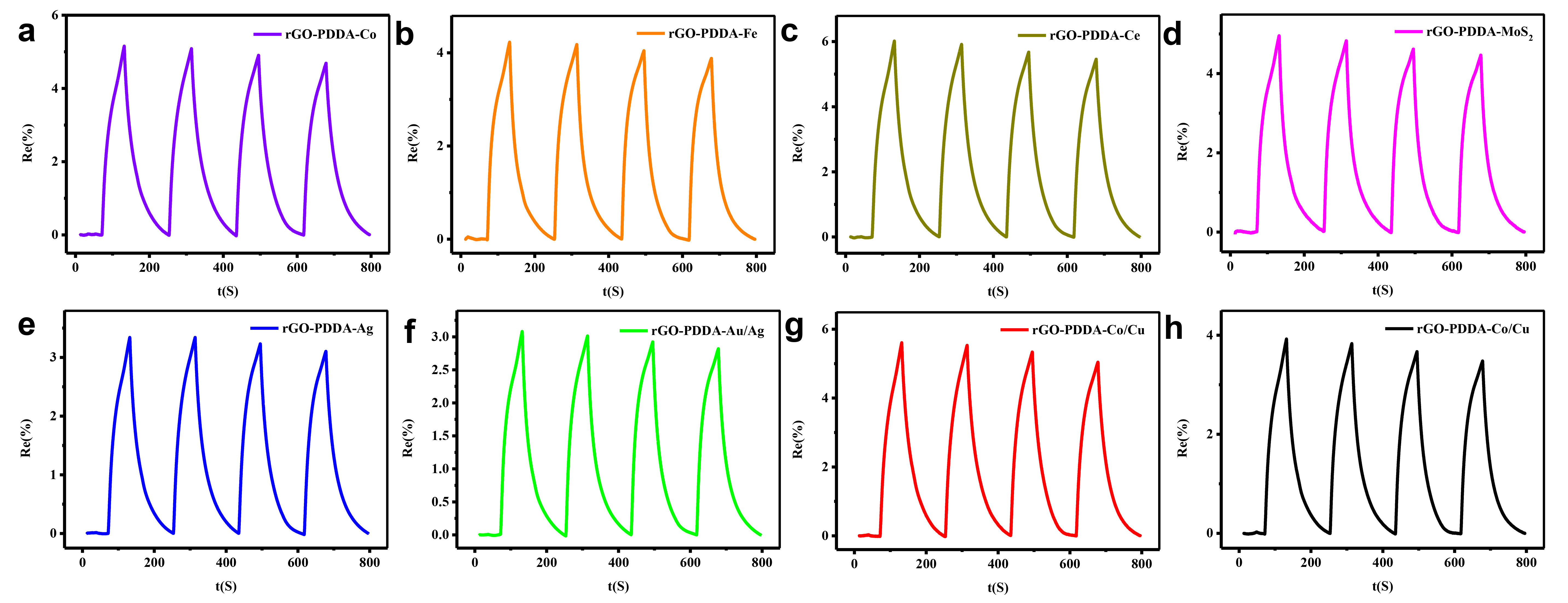


**Figure S10.** Response dynamic curves of rGO-PDDA-M sensors array toward 4% CO_2_.


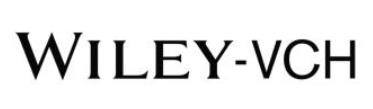


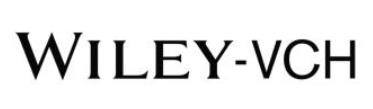


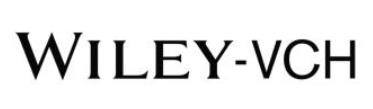


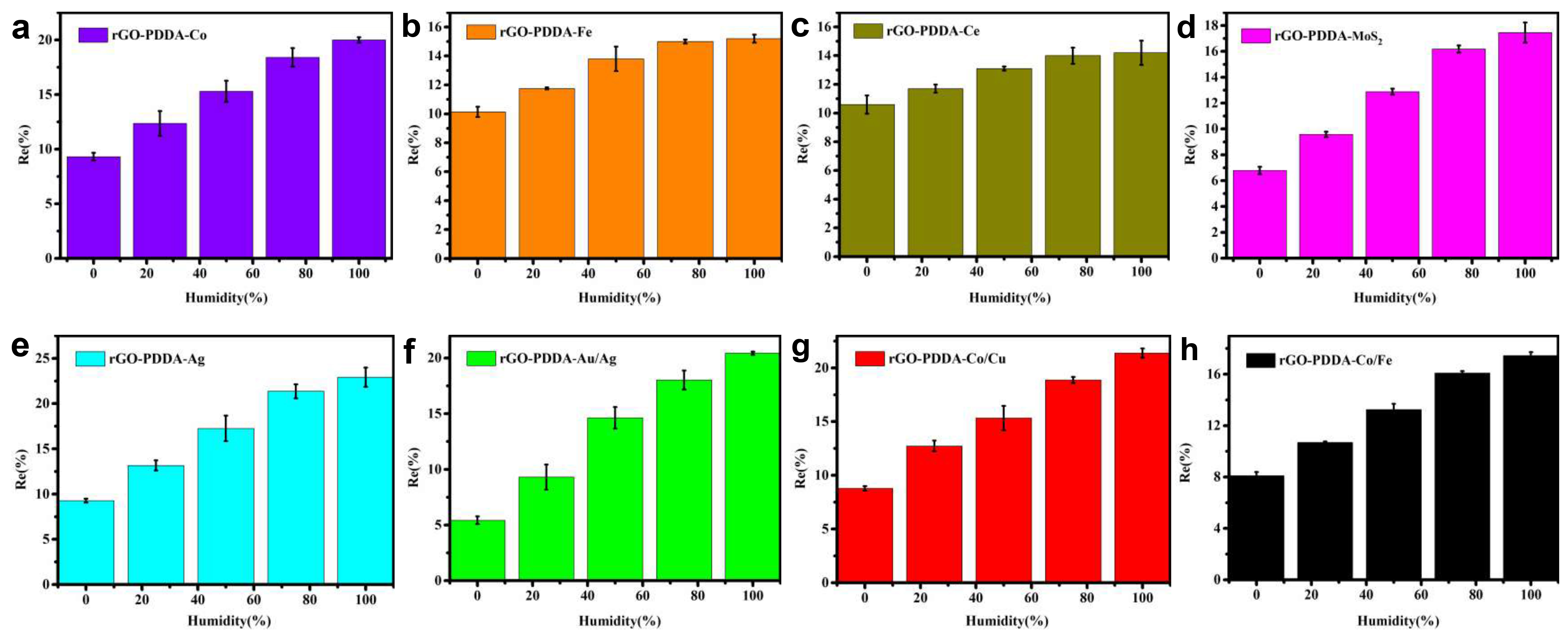


**Figure S11.** (a-h) Response values of rGO-PDDA-M sensor array at different relative humidity. The relative humidity was adjusted by different saturated salt solutions.


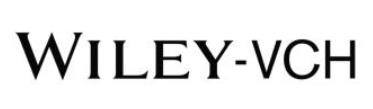


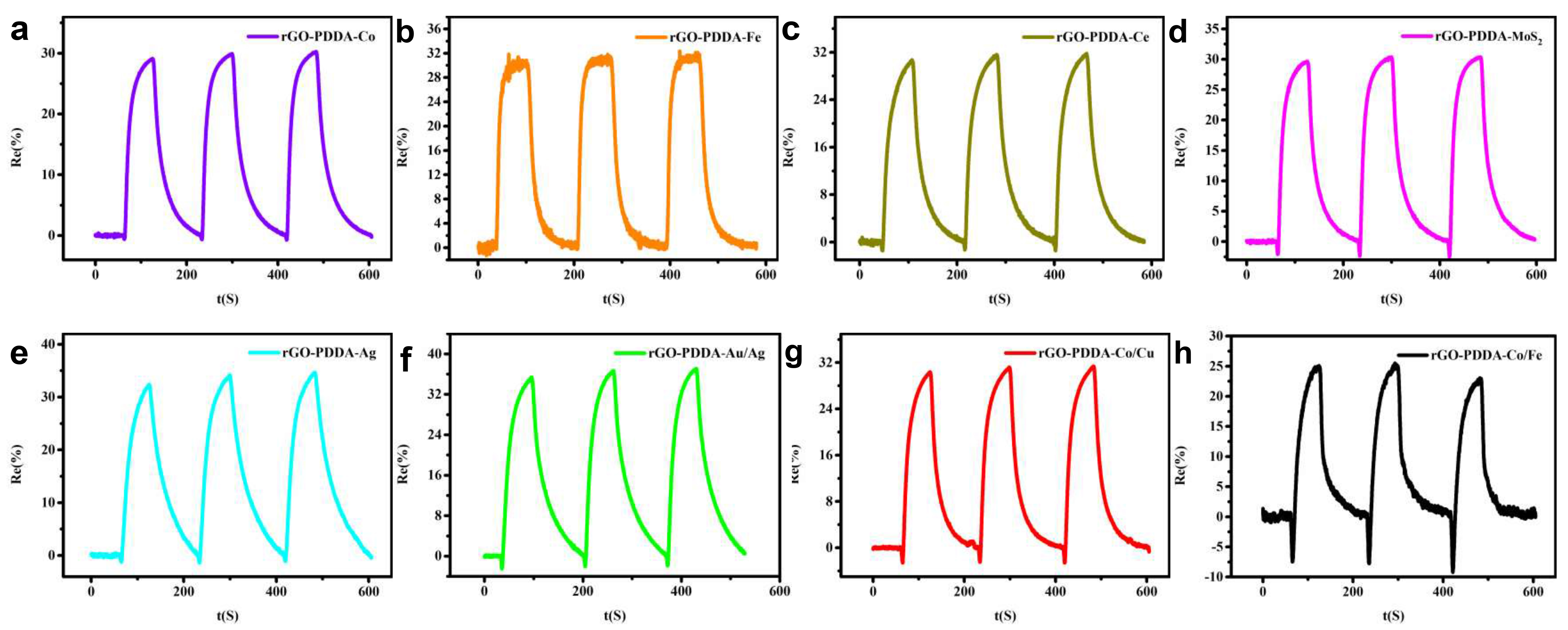


**Figure S12.** Response dynamic curves of rGO-PDDA-M sensors array toward a healthy volunteer 's EB without any pre-treatment.


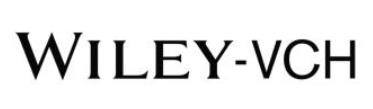


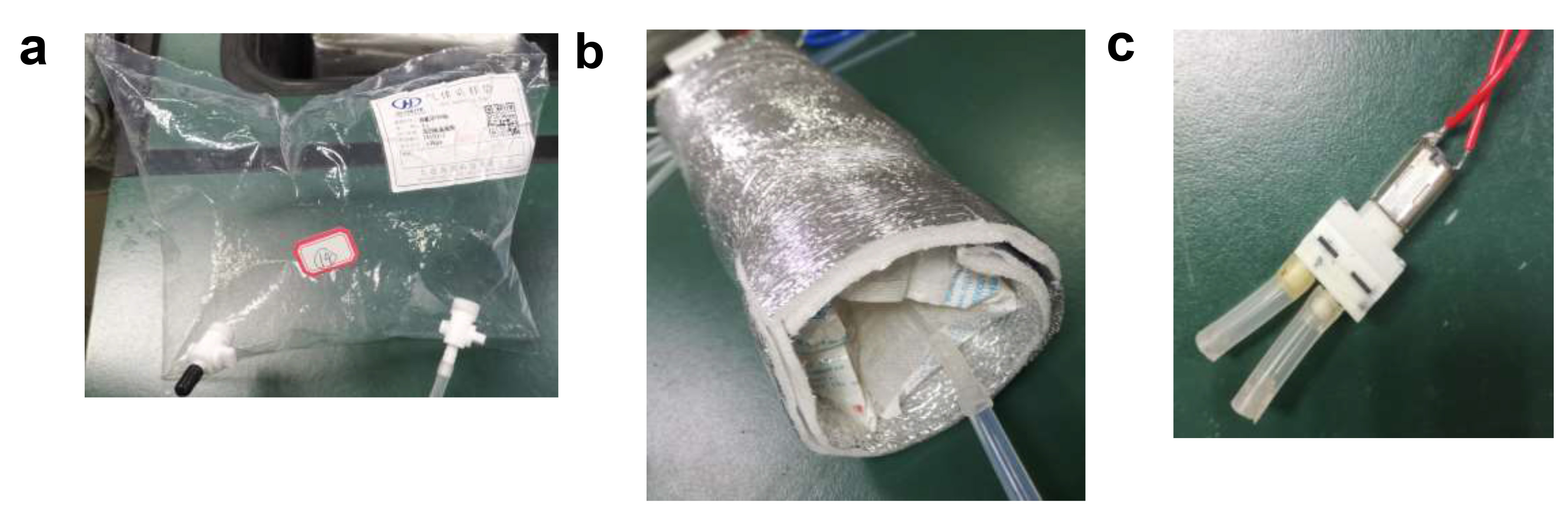


**Figure S13.** (a) Gas sampling bag. (b) Simple condensing unit. (c) Micro pump.


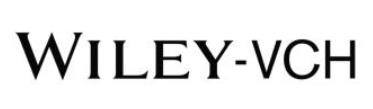


**
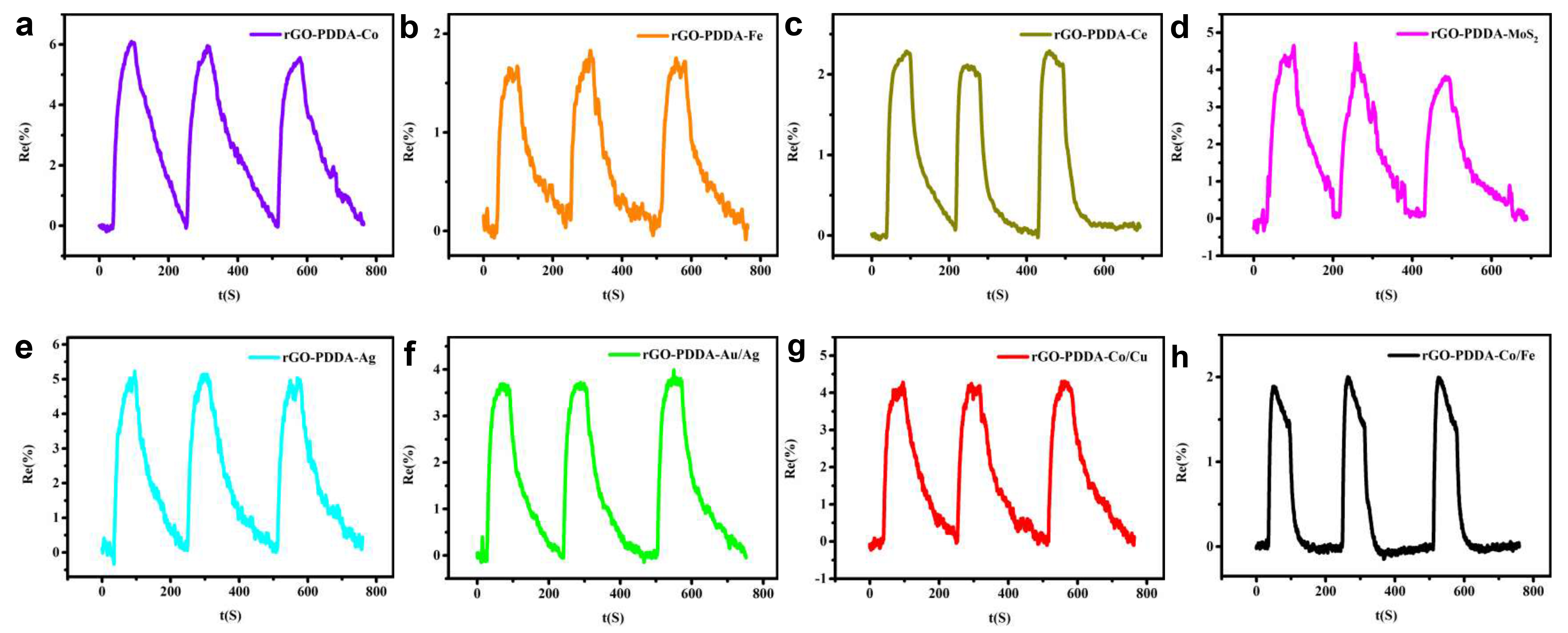
**

**Figure S14.** Response dynamic curves of rGO-PDDA-M sensors array toward a healthy volunteer 's EB after condensation process.


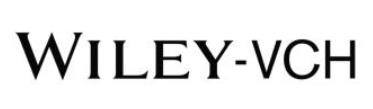


**
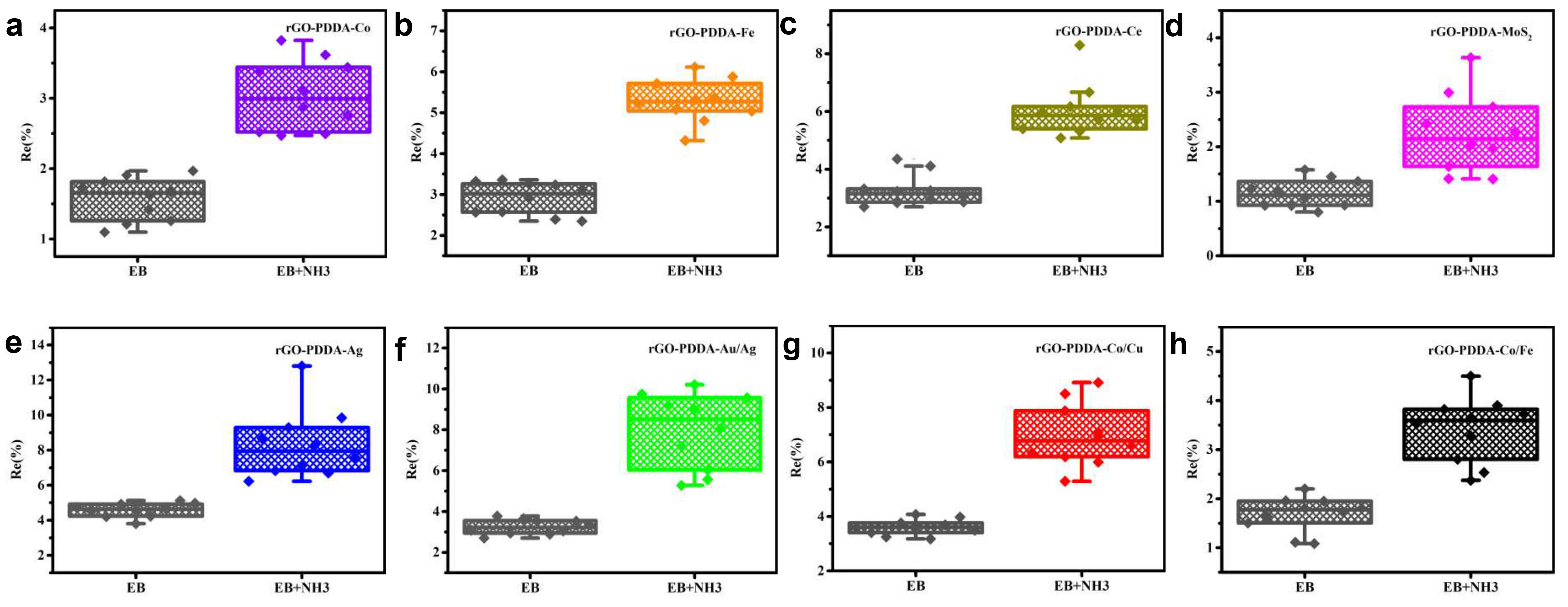
**

**Figure S15.** Box charts of response values for spiked EB samples analysis; (a−h) Responses of rGO-PDDA-Co; rGO-PDDA-Fe; rGO-PDDA-Ce; rGO-PDDA-MoS_2_; rGO-PDDA-Ag; rGO-PDDA-Au/Ag; rGO-PDDA-Co/Cu; and rGO-PDDA-Co/Fe toward EB (blank) (gray) and EB (1ppm NH_3_) (colored) analysis.


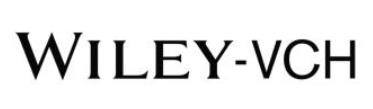


**
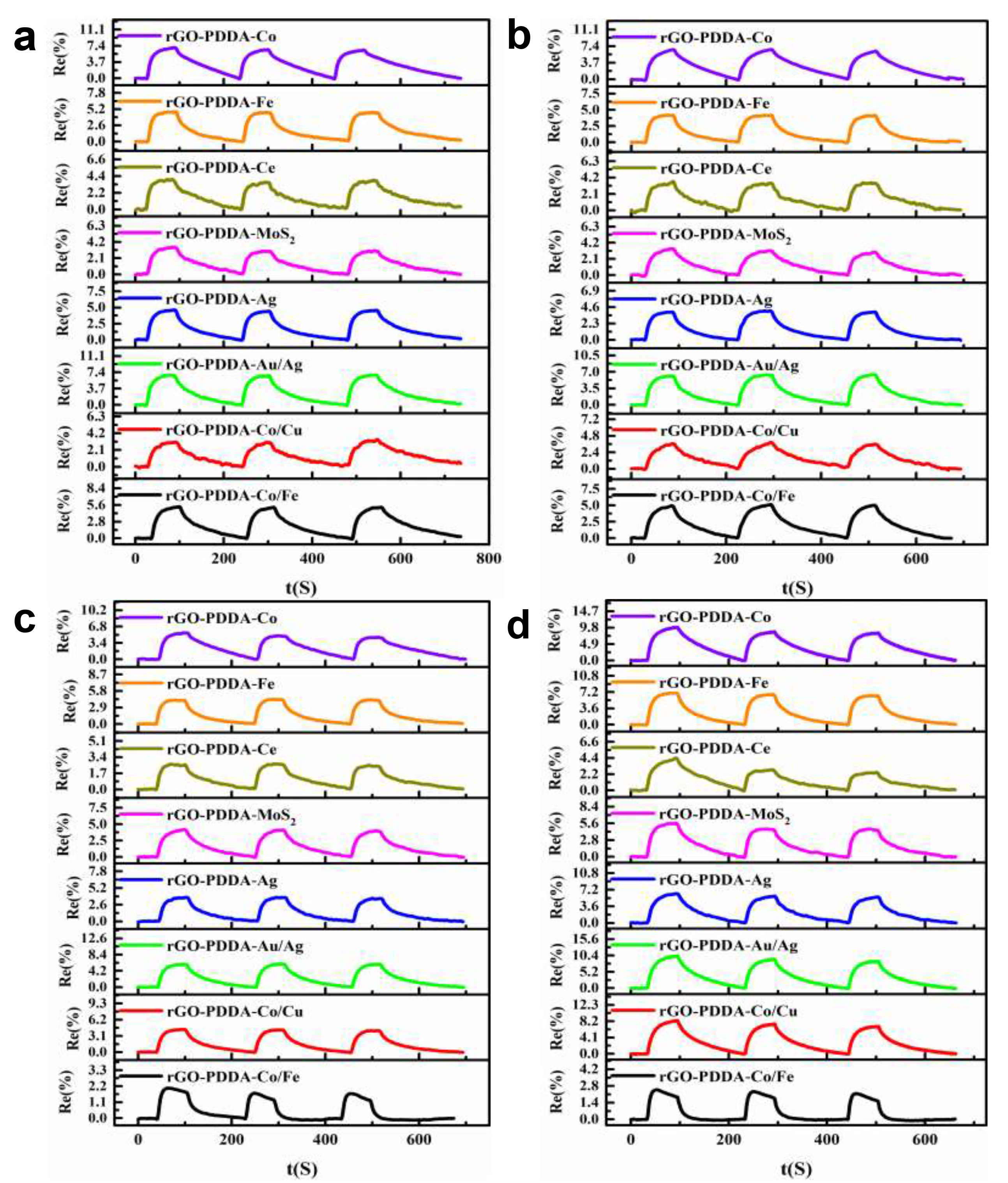
**

**Figure S16.** Typical dynamic response curves of rGO-PDDA-M sensors array in clinical EB samples analysis. (a) and (b) were dynamic responses of EB samples from healthy individuals; (c) and (d) were dynamic response curves of clinical EB samples from HP positives patients.


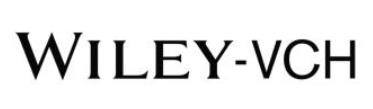

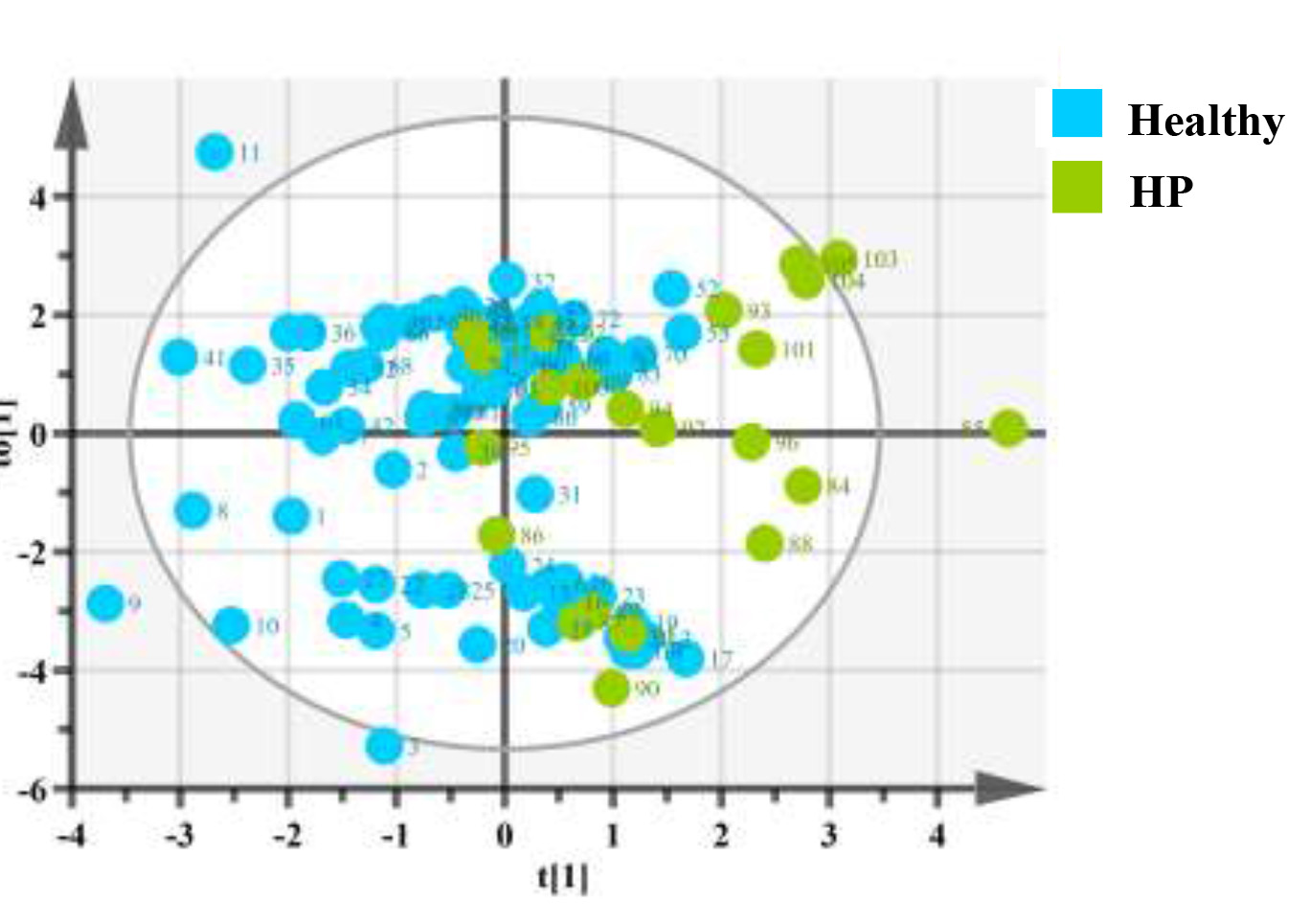


**Figure S17.** The OPLS-DA result of 204 cases clinical EB samples analysis based on the Re% and t_50_ variables of the sensor array.


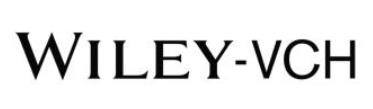


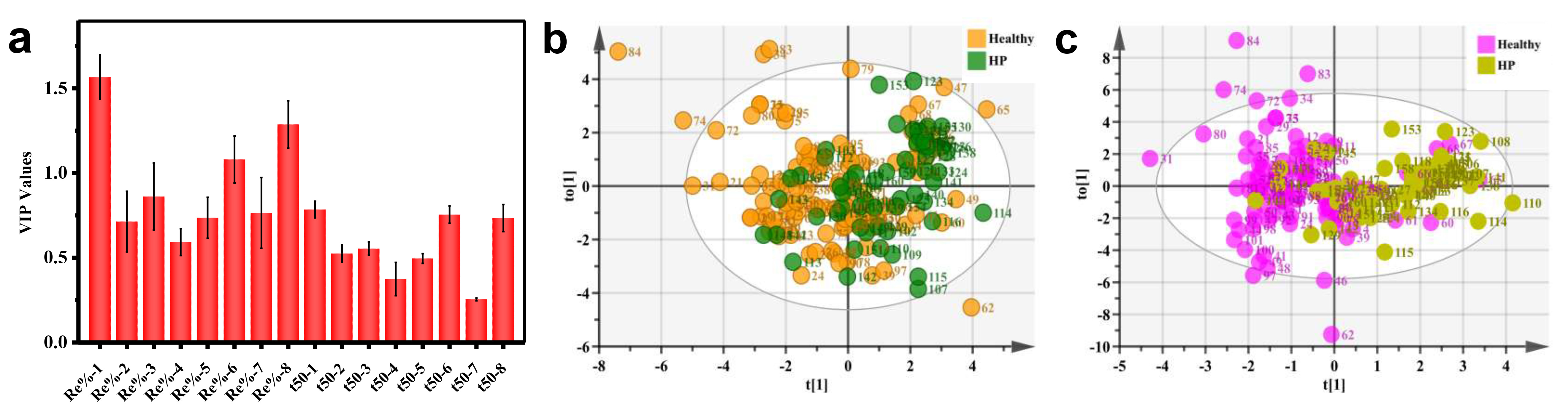


**Figure S18.** (a) The VIP values of Re% and t_50_ variables for 8 composites; 1-8 represents rGO-PDDA-Co; rGO-PDDA-Fe; rGO-PDDA-Ce; rGO-PDDA-MoS_2_; rGO-PDDA-Ag; rGO-PDDA-Au/Ag; rGO-PDDA-Co/Cu; rGO-PDDA-Co/Fe. (b) The OPLS-DA result of 204 cases clinical EB samples based on the t_50_ variable of the sensor array; (c) The OPLS-DA result of 204 cases clinical EB samples based on the Re% variable of the sensor array.


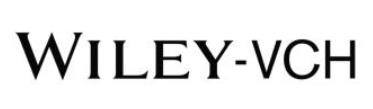


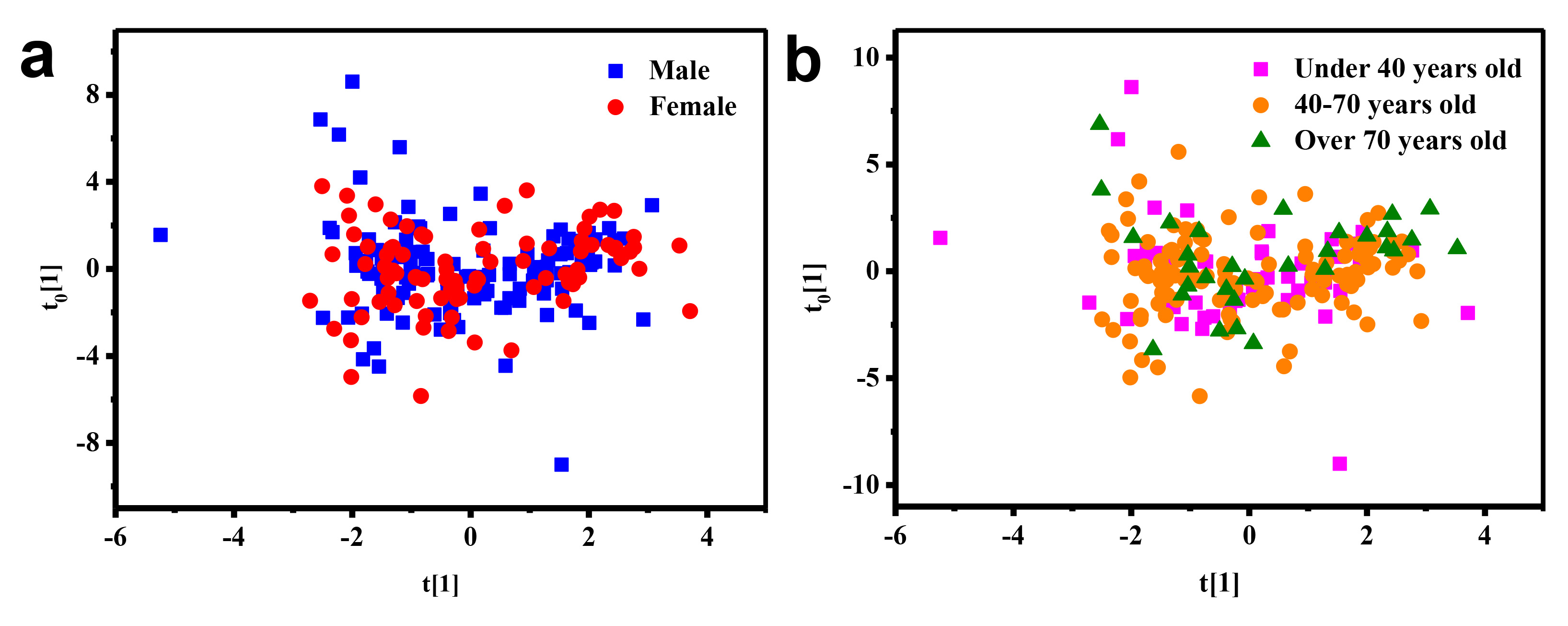


**Figure S19.** The OPLS-DA analysis results of different (a) genders; and (b) ages.


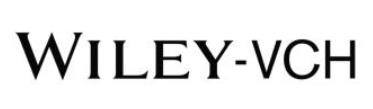


**
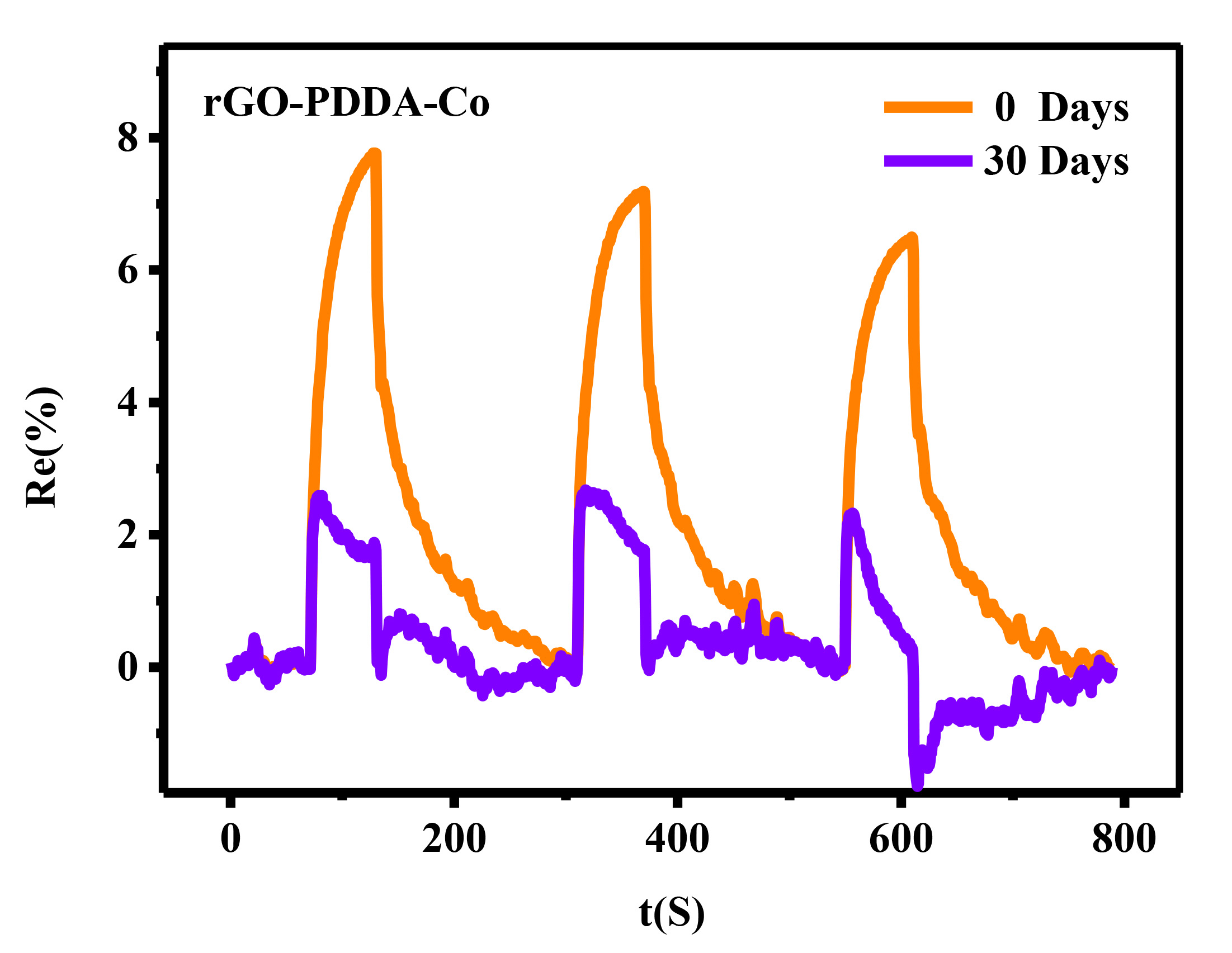
**

**Figure S20.** The response dynamic curves of the rGO-PDDA-Co sensing layer to an EB sample on day 0 and day 30.


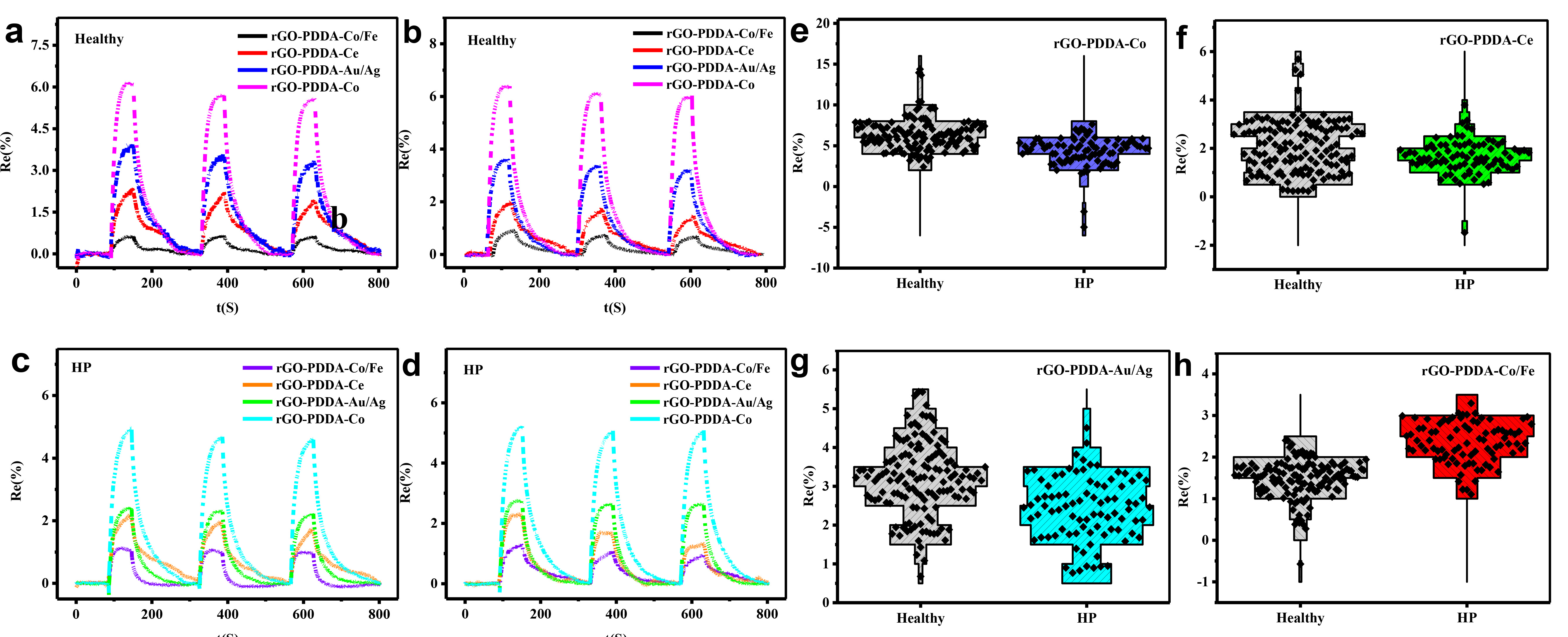

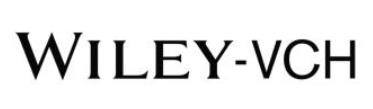


**Figure S21.** The response dynamic curves of different EB samples on the 4-sites micro sensor array. (a-b) Two healthy individuals; (c-d) Two HP-positive individuals. (e-f) The box charts of response values of 225 cases clinic EB samples on 4-sites micro sensor array. (n = 225, α = 0.05, P < 0.05; n is sample size; α is the level of significance test; and the P value is the probability that if the null hypothesis is true, an outcome more extreme than the obtained sample observations will occur.)


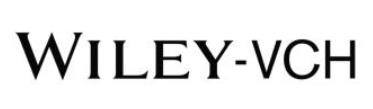

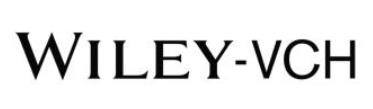

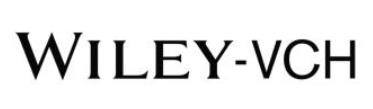


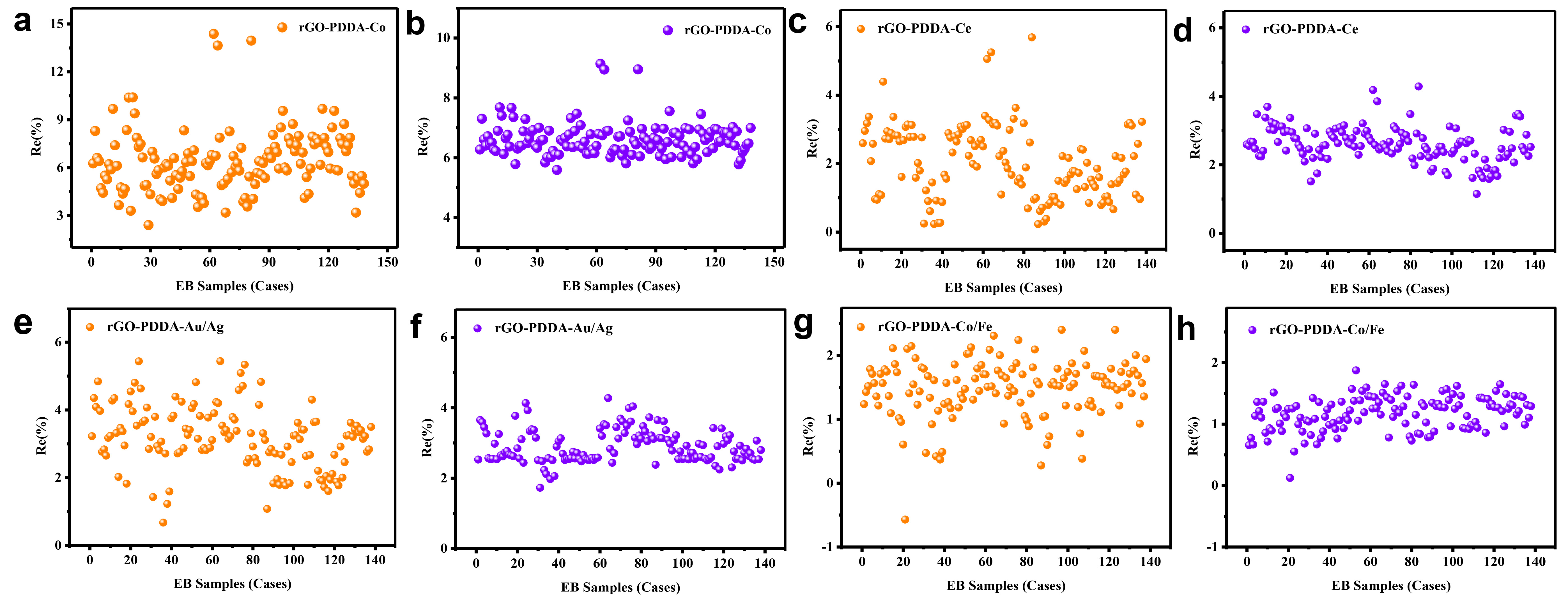


**Figure S22.** The response value fluctuation of 4 sensing layers toward 138 cases healthy individuals (a; c; e; g) before; and (b; d; f; h) after acetone calibration.


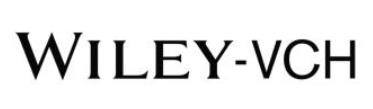


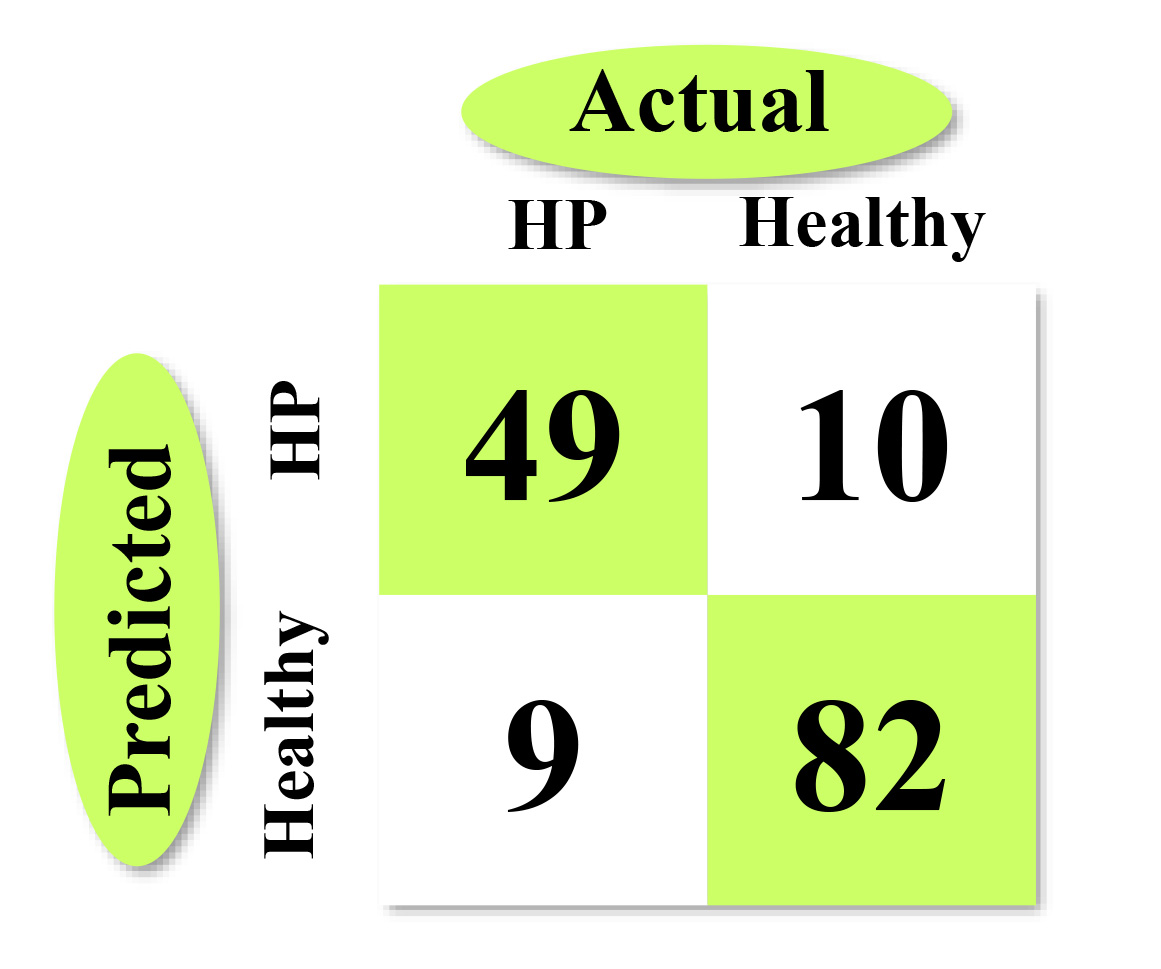


**Figure S23.** Discriminant results of validation set of EB samples based on SVM model.


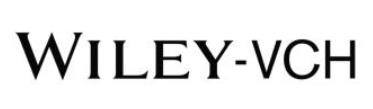


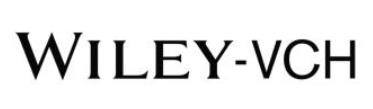


**
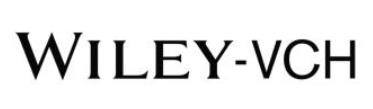
**


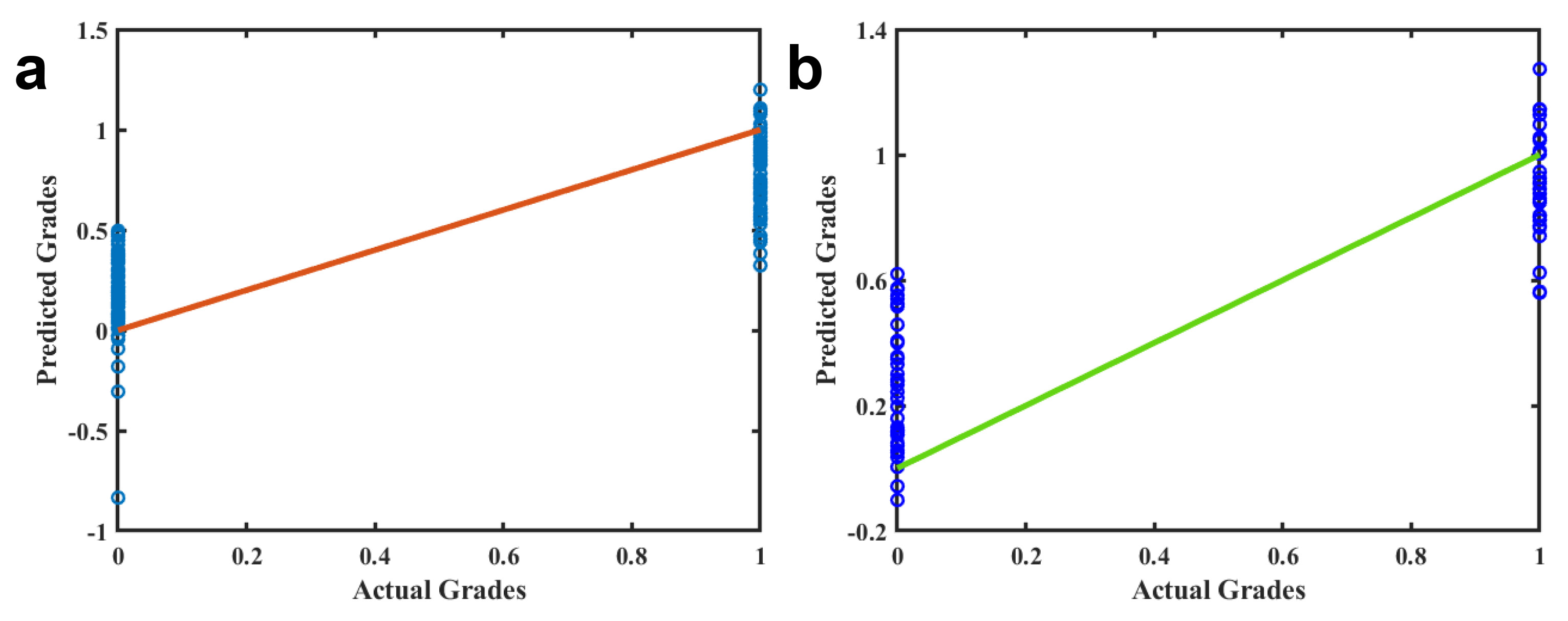


**Figure S24.** The results of Lasso regression analysis of clinical EB samples in (a) training set; and (b) validation set.


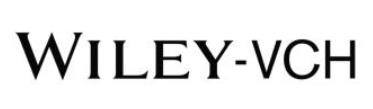
**
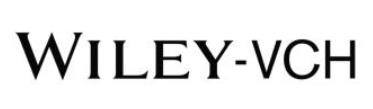
**


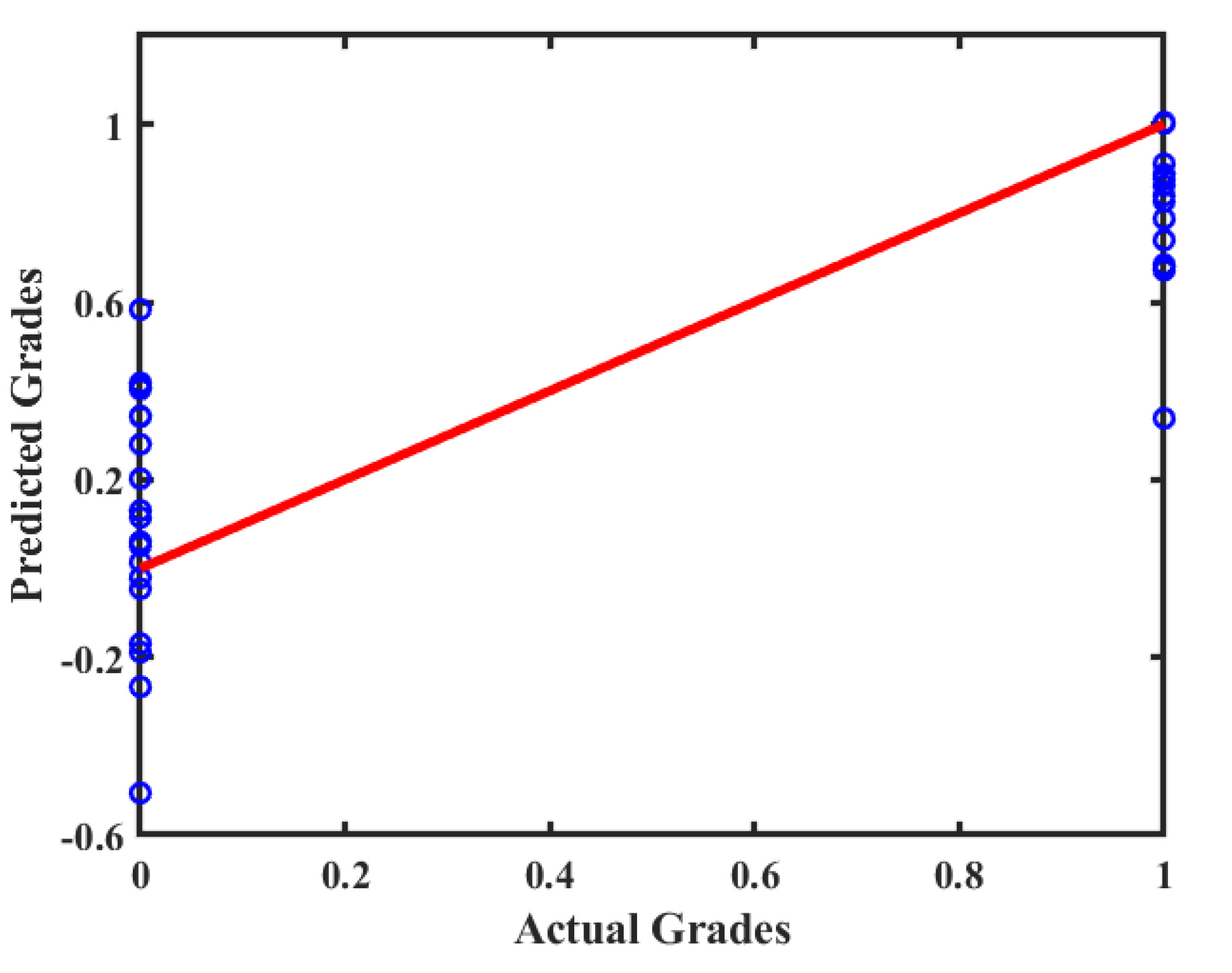


**Figure S25.** The classification results of Lasso model analysis of external test set clinical EB samples.


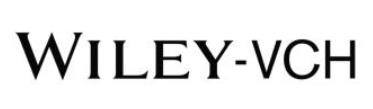
**
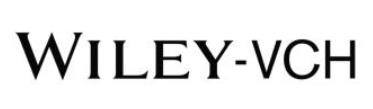
**


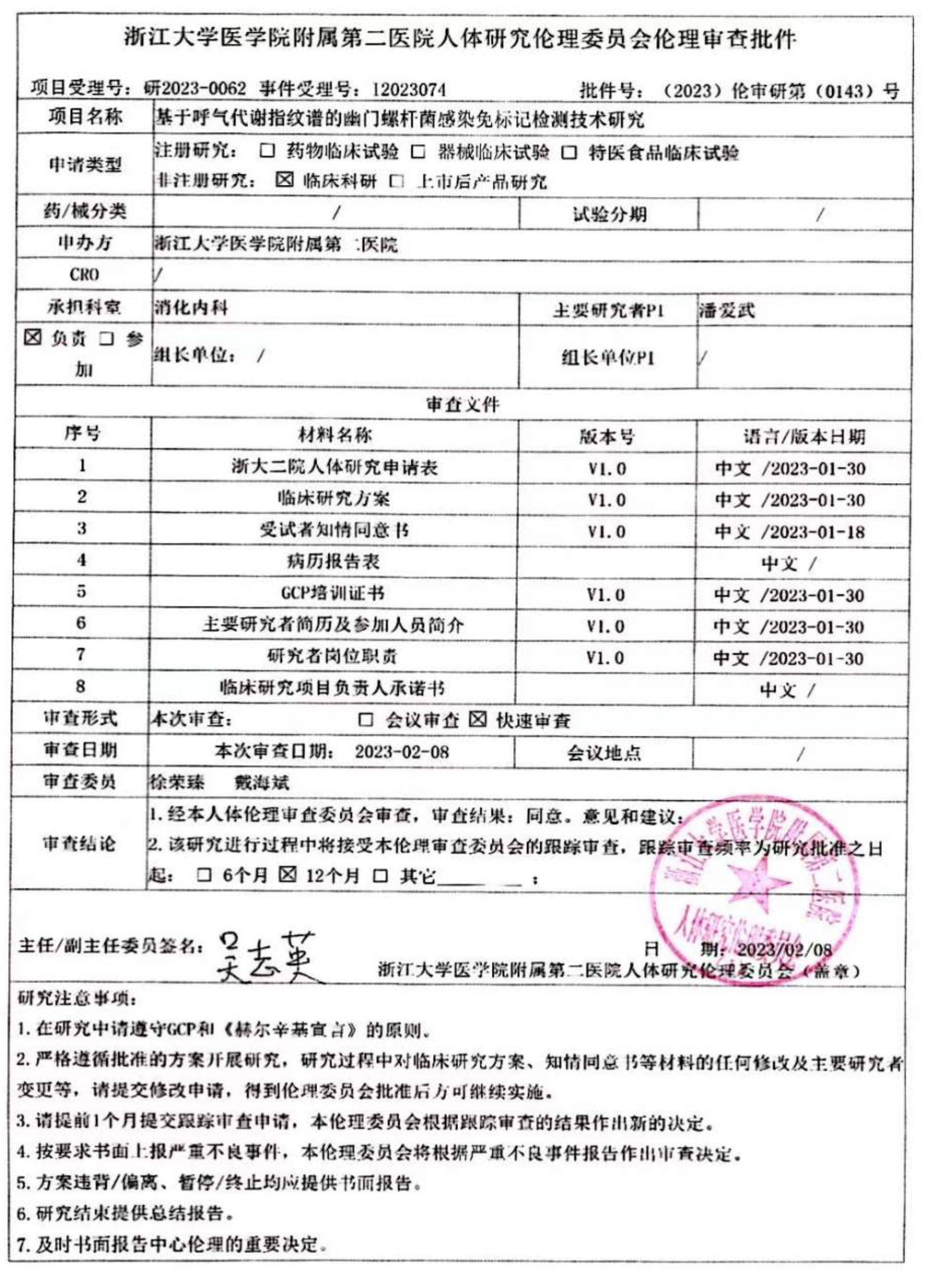


**Figure S26.** The Ethical review from the Second Affiliated Hospital of Zhejiang University School of Medicine.

**
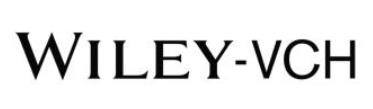
**

**Table S1.** Baseline Noise of different sensing materials.

| Sensing materials | Baseline Noise/N(stedv)% |
| --- | --- |
| rGO | 0.416 |
| rGO-PDDA | 0.118 |
| rGO-PDDA-Co | 0.032 |
| rGO-PDDA-Fe | 0.115 |
| rGO-PDDA-Ce | 0.033 |
| rGO-PDDA-Cu | 0.214 |
| rGO-PDDA-MoS_2_ | 0.097 |
| rGO-PDDA-Au | 0.104 |
| rGO-PDDA-Ag | 0.077 |
| rGO-PDDA-Au/Ag | 0.045 |
| rGO-PDDA-Co/Cu | 0.038 |
| rGO-PDDA-Co/Ce | 0.054 |
| rGO-PDDA-Co/Fe | 0.089 |
| rGO-PDDA-Cu/Ce | 0.134 |


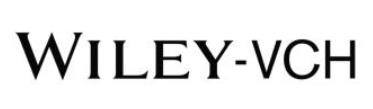


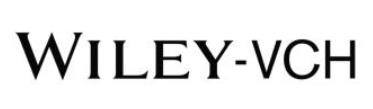

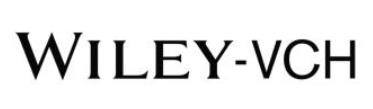

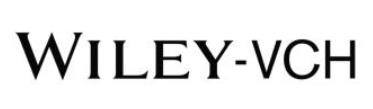


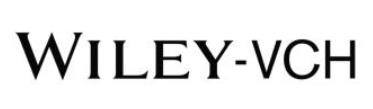


**Table S2.** Langmuir-Hill fitting parameters for rGO-PDDA-M array in acetone, NO, and NH_3_ sensing. ($Re\%=\frac{xyc^{1-z}}{1+yc^{1-z}}$)

| Target gases | Sites | Langmuir-Hill fitting parameters | | | |
| --- | --- | --- | --- | --- | --- |
|  |  | x | y | z | adj. R^2^ |
| Acetone | rGO-PDDA-Co | 10.16558 | 0.06669 | -0.96823 | 0.98023 |
|  | rGO-PDDA-Fe | 9.63521 | 0.06275 | -1.05531 | 0.98205 |
|  | rGO-PDDA-Ce | 12.59838 | 0.06796 | -0.8151 | 0.97384 |
|  | rGO-PDDA-MoS_2_ | 9.99212 | 0.06856 | -0.96823 | 0.97947 |
|  | rGO-PDDA-Ag | 10.98003 | 0.06889 | -0.87605 | 0.97833 |
|  | rGO-PDDA-Au | 8.73923 | 0.05501 | -1.15322 | 0.98446 |
|  | rGO-PDDA-Co/Cu | 11.48407 | 0.0701 | -0.82149 | 0.97587 |
|  | rGO-PDDA-Co/Fe | 9.30848 | 0.05894 | -1.02545 | 0.97975 |
| NO | rGO-PDDA-Co | 10.48762 | 10.78716 | -1.06002 | 0.9911 |
|  | rGO-PDDA-Fe | 11.35079 | 11.45698 | -1.07899 | 0.98948 |
|  | rGO-PDDA-Ce | 12.78433 | 8.50396 | -0.89966 | 0.98429 |
|  | rGO-PDDA-MoS_2_ | 7.58352 | 19.18955 | -1.34847 | 0.97857 |
|  | rGO-PDDA-Ag | 10.4666 | 10.63797 | -1.15852 | 0.96561 |
|  | rGO-PDDA-Au | 6.79396 | 9.62568 | -0.94781 | 0.97398 |
|  | rGO-PDDA-Co/Cu | 10.01277 | 11.14015 | -1.12808 | 0.99133 |
|  | rGO-PDDA-Co/Fe | 9.37625 | 10.35087 | -1.01621 | 0.99783 |
|  | rGO-PDDA-Co | 8.56007 | 2.66073 | -0.60303 | 0.98538 |
|  | rGO-PDDA-Fe | 7.76932 | 2.76125 | -0.55293 | 0.99368 |
|  | rGO-PDDA-Ce | 8.511 | 2.5244 | -0.57898 | 0.98962 |
| NH_3_ | rGO-PDDA-MoS_2_ | 8.26185 | 2.17154 | -0.54595 | 0.98534 |
|  | rGO-PDDA-Ag | 10.14608 | 2.17715 | -0.50979 | 0.97807 |
|  | rGO-PDDA-Au | 8.696 | 2.71827 | -0.60877 | 0.99104 |
|  | rGO-PDDA-Co/Cu | 8.12949 | 2.51371 | -0.74897 | 0.98022 |
|  | rGO-PDDA-Co/Fe | 8.44681 | 2.08398 | -0.47282 | 0.98772 |


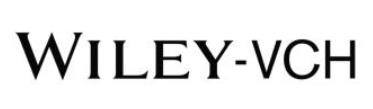

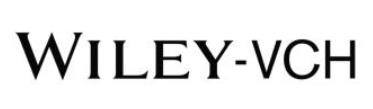

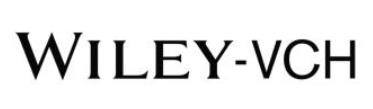


**Table S3.** Linear fitting parameters for rGO-PDDA-M array in isoprene sensing. ($Re\%=a+b*c$)

| Target gases | Sites | Linear fitting parameters | | |
| --- | --- | --- | --- | --- |
|  |  | a | b | adj. R^2^ |
| Isoprene | rGO-PDDA-Co | 0.53616 | -0.08249 | 0.99455 |
|  | rGO-PDDA-Fe | 0.4763 | -0.18597 | 0.99399 |
|  | rGO-PDDA-Ce | 0.53672 | -0.05076 | 0.99454 |
|  | rGO-PDDA-MoS_2_ | 0.48445 | -0.11922 | 0.99395 |
|  | rGO-PDDA-Ag | 0.53447 | -0.13376 | 0.99404 |
|  | rGO-PDDA-Au | 0.44359 | -0.14645 | 0.99512 |
|  | rGO-PDDA-Co/Cu | 0.5858 | -0.03431 | 0.99489 |
|  | rGO-PDDA-Co/Fe | 0.4709 | 0.05661 | 0.98979 |

**Table S4.** Gas concentrations in EB of healthy and *Helicobacter pylori* patients
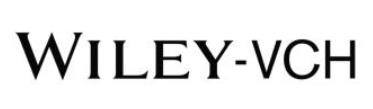
and LOD of the rGO-PDDA-M sensor array.

| Gases | Concentration in healthy people | Concentration in *Helicobacter pylori* patients | LOD of the rGO-PDDA-M sensor array (3δ/S) |  | Ref. |
| --- | --- | --- | --- | --- | --- |
| NO | ~14 ppb | ~27 ppb | 6 ppb |  | [1] |
| NH_3_ | ~383 ppb | ~1245 ppb | 16 ppb |  | [2] |
| isoprene | ~55-121 ppb | ~218 ppb | 190 ppb |  | [3]-[4] |
| Acetone | ~300-900 ppb | has not yet been reported | 68 ppb |  | [5] |


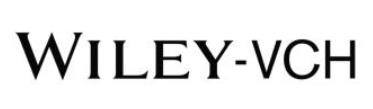


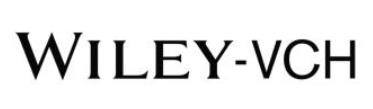


**Table S5.** t_50_ of rGO-PDDA-M sensor array sites for different analytes.

|  | **rGO-PDDA-Co/Fe** | **rGO-PDDA-Co/Cu** | **rGO-PDDA-Au/Ag** | **rGO-PDDA-Ag** | **rGO-PDDA- MoS_2_** | **rGO-PDDA-Ce** | **rGO-PDDA-Fe** | **rGO-PDDA-Co** |
| --- | --- | --- | --- | --- | --- | --- | --- | --- |
| **Ace(1ppm)** | **3.9** | **4.4** | **5.5** | **4.2** | **6** | **4.1** | **5.8** | **4.7** |
| **Ace(2ppm)** | **5.1** | **5.5** | **6.6** | **5.4** | **7.2** | **5.3** | **6.9** | **5.8** |
| **Ace(3ppm)** | **6.6** | **7.2** | **8.3** | **7.1** | **9** | **7** | **8.7** | **7.8** |
| **Ace(5ppm)** | **7.9** | **9.5** | **11.7** | **9.2** | **12.8** | **8.5** | **12.2** | **9.9** |
| **Ace(10ppm)** | **6.8** | **7.5** | **9.2** | **7.3** | **9.7** | **6.9** | **9.5** | **7.7** |
| **Ace(1ppm)** | **4.7** | **5.4** | **6.2** | **5** | **6.7** | **5** | **6.2** | **5.1** |
| **Ace(2ppm)** | **4.9** | **5.3** | **6.3** | **5.2** | **6.8** | **5** | **6.5** | **5.5** |
| **Ace(3ppm)** | **7.4** | **8.1** | **9.2** | **7.8** | **9.7** | **7.7** | **9.3** | **8.5** |
| **Ace(5ppm)** | **8.7** | **10.3** | **12.2** | **10** | **13.2** | **9** | **12.7** | **10.6** |
| **Ace(10ppm)** | **7.9** | **8.4** | **10.2** | **8.2** | **10.5** | **8** | **10.4** | **8.8** |
| **Ace(1ppm)** | **3.6** | **4.2** | **5.1** | **3.9** | **5.7** | **3.8** | **5.4** | **4.3** |
| **Ace(2ppm)** | **4.9** | **5.3** | **6.3** | **5.2** | **6.8** | **5** | **6.5** | **5.5** |
| **Ace(3ppm)** | **6.4** | **7** | **8** | **6.7** | **8.7** | **6.6** | **8.3** | **7.3** |
| **Ace(5ppm)** | **7.5** | **9.2** | **11.3** | **9** | **12.3** | **8** | **11.8** | **9.5** |
| **Ace(10ppm)** | **6.7** | **7.4** | **9.1** | **7.1** | **9.5** | **6.9** | **9.2** | **7.8** |
| **NO(0.1ppm)** | **8.5** | **10.6** | **4.8** | **18.6** | **4.2** | **16.7** | **20.6** | **19.3** |
| **NO(0.2ppm)** | **13.6** | **16.8** | **8.2** | **29.3** | **7.9** | **26.6** | **33.1** | **31.3** |
| **NO(0.3ppm)** | **12.6** | **15.2** | **7.1** | **29.7** | **6.7** | **25.3** | **33.4** | **31** |
| **NO(0.5ppm)** | **20.4** | **23.9** | **14.6** | **38.3** | **13.4** | **35.2** | **47.3** | **40** |
| **NO(1ppm)** | **53** | **61.3** | **33.7** | **96.4** | **31.8** | **90** | **99.4** | **97.3** |
| **NO(0.1ppm)** | **13.1** | **15.8** | **8.4** | **21.5** | **7.3** | **19.4** | **23.5** | **22.2** |
| **NO(0.2ppm)** | **20.9** | **23.5** | **14.6** | **33.6** | **12.4** | **30.6** | **41.2** | **35** |
| **NO(0.3ppm)** | **19.8** | **22** | **13** | **35** | **11.5** | **31.5** | **41.1** | **36.7** |
| **NO(0.5ppm)** | **29** | **34.5** | **22.8** | **46.9** | **21.4** | **44.9** | **55.7** | **48.5** |
| **NO(1ppm)** | **65** | **74** | **44** | **108** | **41.5** | **100** | **112.6** | **109.4** |
| **NO(0.1ppm)** | **11.4** | **13.7** | **6.4** | **19.6** | **5.8** | **17.7** | **20.6** | **19.9** |
| **NO(0.2ppm)** | **17.5** | **20.5** | **11** | **31.5** | **10** | **28.8** | **39.1** | **33.1** |
| **NO(0.3ppm)** | **16.3** | **19** | **10** | **31** | **9** | **28** | **37** | **33** |
| **NO(0.5ppm)** | **25.4** | **28.7** | **19.6** | **42** | **16.5** | **39.4** | **51** | **44** |
| **NO(1ppm)** | **59** | **67.8** | **39** | **101** | **36.4** | **95.5** | **106** | **102** |
| **Iso(2ppm)** | **8.8** | **5.9** | **3.3** | **4** | **6.1** | **7.9** | **3.9** | **5.5** |
| **Iso(5ppm)** | **11.9** | **7.4** | **3.9** | **6** | **7.9** | **10.5** | **5** | **7.1** |
| **Iso(8ppm)** | **13.1** | **8.8** | **4.2** | **7.1** | **8.9** | **11.8** | **6.6** | **8.8** |
| **Iso(12ppm)** | **13.8** | **9** | **4.8** | **7.9** | **9.3** | **12** | **7.4** | **9** |
| **Iso(15ppm)** | **14** | **10** | **6** | **9.1** | **10.6** | **12.9** | **8.5** | **9.2** |
| **Iso(2ppm)** | **14.8** | **11.8** | **5.6** | **10** | **12.2** | **13.6** | **8.1** | **10.2** |
| **Iso(5ppm)** | **17.4** | **13.2** | **7.9** | **12.6** | **13.9** | **17.3** | **10.2** | **12.9** |
| **Iso(8ppm)** | **19.6** | **14.5** | **8.7** | **13.2** | **15.5** | **17.4** | **11.9** | **14.3** |
| **Iso(12ppm)** | **20** | **15.4** | **10.8** | **14.7** | **16** | **19.2** | **13.8** | **15.5** |
| **Iso(15ppm)** | **22** | **16.1** | **10** | **15** | **17** | **20.5** | **14** | **15.7** |
| **Iso(2ppm)** | **11.5** | **7.1** | **4.2** | **5.7** | **8.4** | **10** | **5** | **7** |
| **Iso(5ppm)** | **13.4** | **9.2** | **5.3** | **7.7** | **10** | **12.7** | **7.8** | **9.4** |
| **Iso(8ppm)** | **14.1** | **10.7** | **6.2** | **8.8** | **11.8** | **13** | **8.5** | **10.3** |
| **Iso(12ppm)** | **15.7** | **11** | **6.6** | **9.2** | **12.4** | **14.5** | **9** | **11** |
| **Iso(15ppm)** | **16** | **12** | **7.2** | **10.5** | **13** | **15** | **10** | **11.5** |
| **NH_3_(0.2ppm)** | **7.9** | **9** | **9.8** | **8.7** | **9** | **9.3** | **3.6** | **9.3** |
| **NH_3_(0.3ppm)** | **9** | **10.2** | **11** | **10** | **10.5** | **10.9** | **3.8** | **10.9** |
| **NH_3_(0.5ppm)** | **8.1** | **9.3** | **10.3** | **9.2** | **9.6** | **10.1** | **4.1** | **9.2** |
| **NH_3_(1ppm)** | **9.1** | **10.5** | **11.5** | **10** | **10.6** | **11** | **4.3** | **10.5** |
| **NH_3_(2ppm)** | **8** | **9** | **10.6** | **8.6** | **9.4** | **9.6** | **5.5** | **9.4** |
| **NH_3_(0.2ppm)** | **9** | **10.7** | **11.9** | **10.1** | **10.7** | **10.9** | **4.7** | **10.6** |
| **NH_3_(0.3ppm)** | **10.5** | **12** | **13.5** | **11.7** | **12.1** | **12.2** | **5.6** | **12.2** |
| **NH_3_(0.5ppm)** | **9.2** | **11.1** | **12.2** | **10.5** | **11** | **11.3** | **6.2** | **11.1** |
| **NH_3_(1ppm)** | **10** | **12.3** | **13** | **11.3** | **12.3** | **12.5** | **6.7** | **12.4** |
| **NH_3_(2ppm)** | **9.3** | **11** | **12.1** | **10** | **11.2** | **11.8** | **7.1** | **11.6** |
| **NH_3_(0.2ppm)** | **6.5** | **8.6** | **9** | **6.7** | **8.6** | **8.9** | **3.1** | **9.1** |
| **NH_3_(0.3ppm)** | **7.8** | **9.2** | **10.3** | **7.9** | **9.4** | **10.3** | **3.7** | **10.1** |
| **NH_3_(0.5ppm)** | **7.4** | **8.3** | **9.1** | **7.7** | **8.2** | **9** | **4** | **8.9** |
| **NH_3_(1ppm)** | **8.5** | **9.7** | **10.2** | **8.5** | **9.8** | **10** | **4.3** | **9.9** |
| **NH_3_(2ppm)** | **6.9** | **8.4** | **9.4** | **7** | **8.5** | **9.2** | **4.7** | **9** |


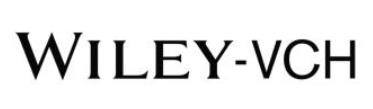


**Video S1.** The video of EB sample detection on the e-nose prototype.

**Reference:**

[1] J. Kasperski, M. Wyszynskai, S. Kustra, E. Czecior, M. Misiolek, A. Kasperska-Zajac, *European Journal of Inflammation* **2013**, 11(1), 279-282

[2] I. Bayrakli, A. Turkmen, M. Cem Kockar, *Appl Spectrosc* **2016**, *70* (8), 1269.

[3] Z. Q. Xu, Y. Y. Broza, R. Ionsecu, U. Tisch, L. Ding, H. Liu, Q. Song, Y. Y. Pan, F. X. Xiong, K. S. Gu, G. P. Sun, Z. D. Chen, M. Leja, H. Haick, *Br J Cancer* **2013**, *108* (4), 941.

[4] A.M. Diskin, P. Spanˇel, D. Smith, *Physiological measurement* **2003**, 24(1), 107.

[5] J. Lee, K.L Chan, J.P. Hyung, S.S.C. Hyunjoon, D.S. Lee, *ACS applied materials & interfaces***2020**, 12(31), 35688-35697.
